# Views and experience of breastfeeding in public: A qualitative systematic review

**DOI:** 10.1111/mcn.13407

**Published:** 2022-08-01

**Authors:** Aimee Grant, Bethan Pell, Lauren Copeland, Amy Brown, Rebecca Ellis, Delyth Morris, Denitza Williams, Rhiannon Phillips

**Affiliations:** ^1^ Centre for Trials Research Cardiff University Cardiff UK; ^2^ Centre for Lactation, Infant Feeding and Translational Research Swansea University Swansea UK; ^3^ DECIPHer Cardiff University Cardiff UK; ^4^ Division of Population Medicine Cardiff University Cardiff UK; ^5^ Subject Librarian, Dental and Medicine Cardiff University Cardiff UK; ^6^ Cardiff School of Sport and Health Sciences Cardiff Metropolitan University Cardiff UK

**Keywords:** breastfeeding, breastfeeding in public, infant feeding, sexualisation of breasts, shaming, stigma

## Abstract

Breastfeeding rates in many Global North countries are low. Qualitative research highlights that breastfeeding in public is a particular challenge, despite mothers often having the legal right to do so. To identify barriers and facilitators, we systematically searched the qualitative research from Organisation for Economic Co‐operation and Development countries relating to breastfeeding in public spaces from 2007 to 2021. Data were analysed using the Thematic Synthesis technique. The review was registered with PROSPERO (registration number: CRD42017081504). Database searching identified 3570 unique records. In total, 74 papers, theses, or book chapters, relating to 71 studies, were included, accounting for over 17,000 mothers. Overall, data quality was high. Our analysis identified that five core factors influenced mothers' thought processes and their breastfeeding in public behaviour: *legal system; structural (in)equality; knowledge; beliefs and the social environment*. Macro‐level factors relating to legislation and inequality urgently require redress if breastfeeding rates are to be increased. Widespread culture change is also required to enhance knowledge, change hostile beliefs and thus the social environment in which mother/infant dyads exist. In particular, the sexualisation of breasts, disgust narratives and lack of exposure among observers to baby‐led infant feeding patterns resulted in beliefs which created a stigmatising environment. In this context, many mothers felt unable to breastfeed in public; those who breastfed outside the home were usually highly self‐aware, attempting to reduce their exposure to conflict. Evidence‐based theoretically informed interventions to remove barriers to breastfeeding in public are urgently required.

## Key messages

- In OECD countries, there was limited evidence that women routinely breastfed outside of the home; for those who did, this experience was often uncomfortable.
- Legal protections for breastfeeding in public, where present, were not widely known and appeared to be under‐enforced.
- Mothers were aware of stigma relating to breastfeeding in public and feared conflict with strangers. This fear was not felt evenly, with mothers who were young, poor, and from marginalised ethnicities reporting increased surveillance and stigma.
- There is limited and dichotomous evidence relating to partners, family, and friends' roles in supporting mothers to breastfeed in public.
- Observers of breastfeeding in public had poor understanding of normal infant feeding behaviour and the associated *need* to breastfeed in public spaces. They also experienced disgusted reactions to viewing breastfeeding.

## Graphical abstract

Factors influencing mothers' infant feeding in public spaces

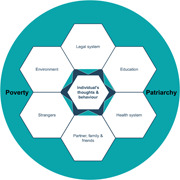

## 1 INTRODUCTION

Increasing breastfeeding rates is a public health policy objective in many developed countries (Rollins et al., 2016). Within many Organisation for Economic Co‐operation and Development (OECD) countries, which we use as a proxy for Global North countries, women have a legal right to breastfeed (Brown, 2021). In some countries, this right is explicitly included in law, for example under the UK Equality Act 2010 and the Republic of Ireland Equal Status Act 2000. In other countries, legal permission to breastfeed in public is implicit, for example, in the Basic Law for the Federal Republic of Germany which protects the rights of parents, and the Canadian Charter of Rights and Freedoms which gives equal status to men and women's freedom. However, an integrative review of evidence has shown that breastfeeding in public is challenging for those who are breastfeeding, with no safe space to breastfeed regularly reported (Hauck et al., 2021). Feeling unable to breastfeed in public spaces (de Jager et al., 2013), or perceiving the neighbourhood as unsafe for children to play in (Peregrino et al., 2018) are known barriers to breastfeeding continuation. Evidence shows that, as well as maternal embarrassment and social discomfort, partners (Andrew & Harvey, 2011) and observers (Henderson et al., 2011) find breastfeeding in public uncomfortable. Furthermore, although not all public places are staffed, where there *are* employees who could help to protect mothers' legal right to breastfeed, they can find it challenging to support breastfeeding mothers (Marsden & Abayomi, 2012) or may be unaware of the law (Alb et al., 2017).

Within the existing integrative review, key challenges to breastfeeding in public were drawn from 27 papers which were represented 12 countries worldwide, including China, Ghana, Romania, Singapore, and Thailand (Hauck et al., 2020). By contrast, our systematic review was restricted to qualitative research on perceptions and experiences of breastfeeding in public spaces within OECD countries, to reduce heterogeneity across findings and shape the design of future interventions aimed at reducing barriers to breastfeeding outside of the home in high‐ and middle‐income countries. Furthermore, whilst Hauck et al. (2020) eliminated 11 of the 38 manuscripts on the basis of quality, we did not exclude articles on the basis of quality as long as their findings contained at least a paragraph of content relating to views and experiences of breastfeeding in public and were therefore felt to have value.

## 2 METHODS

### 2.1 Aim

To undertake a qualitative systematic review investigating barriers and facilitators to breastfeeding in public in OECD countries using the Thematic Synthesis approach (Thomas & Harden, 2008).

### 2.2 Search strategy

We identified the search terms to be included in the review by hand‐searching keywords of relevant papers and terms used in relevant systematic reviews. The search strategy, developed with the support of a specialist librarian (DM), involved two main terms relating to (i) breastfeeding and (ii) public space. A search strategy was developed in Medline (see Appendix A) and was adapted during the searches of other databases.

Following the publication of our protocol on the PROSPERO website, DM searched five electronic databases (Medline via Ovid, Web of Science, EMBASE via Ovid, PsychINFO via Ovid, and CINAHL via EBSCO). We searched a range of databases to reflect the range of academic disciplines (medicine, nursing, allied health professionals and social sciences) contributing to the academic literature in this area, and limited our search to humans. The databases were searched initially for a period of 10 years, to ensure relevance when designing interventions, from 2007 to November 2017, with the searches updated to May 2021 before publication. Evidence reviews identified in database searching were unpicked; that is all papers included within that evidence review were assessed for eligibility. Alongside database searching, we hand‐searched key journals (*Journal of Human Lactation* and *Maternal and Child Nutrition*), publishers (Policy Press, Routledge, SAGE and Pinter and Martin) and Amazon.co.uk for relevant articles and books. All papers included in the review were subjected to forward and backward chaining.

### 2.3 Study selection

Qualitative and mixed methods studies which focused on experiences and views of breastfeeding in public spaces among those living within OECD countries were included. Studies were assessed against pre‐defined inclusion and exclusion criteria.

#### 2.3.1 Inclusion criteria

1. Population
  - Pregnant women and/or mothers (including adoptive and nonbiological mothers)
  - Those who influence breastfeeding in any age of baby or child, including partners, family, friends, and health professionals
  - Members of the public (observers or would be observers of breastfeeding in public)
2. Context
  - Any setting within OECD countries which is open to the public and is outside of the home or homes of friends and family
3. Phenomenon
  - Studies with a focus on preferences, attitudes, and experiences of breastfeeding (to include expressed breastmilk) in public

#### 2.3.2 Exclusion criteria

1. Studies that did not have at least a paragraph of content focused on preferences, attitudes, and experiences of breastfeeding in public
2. Not a qualitative study (i.e. not based on open text survey responses or a qualitative method)
3. Not an OECD country
4. Full text does not exist (including conference abstracts)
5. Full text not available in English language

Two reviewers (AG and either Michael Robling or RE) independently reviewed all titles and abstracts identified through the searches against the inclusion and exclusion criteria. Any inconsistencies were resolved through discussion, and it was not necessary to involve a third reviewer. The full texts of potentially relevant studies were reviewed independently by two reviewers (AG and either BP or RE).

### 2.4 Quality assessment

All included studies were subjected to the CASP critical appraisal checklist (Critical Appraisal Skills Programme, 2018) by one researcher (AG). A sample of 10% of included studies was independently appraised by a second reviewer (LC). Each study was provided with an overall assessment of quality using the categories ‘high’, ‘medium’ and ‘low’, based on the number of criteria fulfilled, an approach which has been utilised in other qualitative syntheses (Woodman et al., 2016). All studies were included in the synthesis regardless of CASP score as long as their findings were felt to be valuable (question 10 on the CASP checklist) due to the heterogeneity of methods and disciplines involved in research on breastfeeding in public. However, quality was used to interpret the relevance of the findings and CASP scores are reported in Table 2. Alongside undertaking the CASP assessment, a data extraction sheet was developed which comprised of demographic characteristics and space for all qualitative findings relating to the review's focus (including within abstracts and appendices). This was to enable study characteristics, critical appraisal and qualitative data to be reviewed together.

### 2.5 Qualitative synthesis: Thematic synthesis

We followed the Thomas and Harden (2008) thematic synthesis procedure: coding text, development of descriptive themes and analytical theme generation. Two researchers (AG and RP) with different disciplinary backgrounds (sociology/health psychology) inductively hand‐coded five of the included papers and met to discuss the codes they had utilised to see if similar codes were developed. There was considerable overlap in how data extracts had been grouped, although different names were assigned to these early codes. Following this, each study document relating to studies from 2007 to 2017 (structured summary, CASP and qualitative findings) was added to NVivo 11 to allow for coding by AG. To reduce bias from relevant data being excluded from the coding framework, each sentence of data which related to breastfeeding in public received at least one code (line‐by‐line coding), and many received more than one code. Where new codes were identified during the analysis, previously coded studies were reviewed and recoded where necessary.

A series of three data analysis meetings were held between (AG, BP, LC, RP and Michael Robling) to refine the development of descriptive themes, enabling the translation of concepts between papers. To promote analytical theme generation within the multi‐disciplinary team, the NVivo analysis file and a ‘summary of themes’ document (containing selected data extracts and a narrative presented within AG's initial thematic coding structure) was reviewed by each researcher independently, who inferred barriers and facilitators to breastfeeding from these descriptive themes, as per thematic synthesis guidance (Thomas & Harden, 2008). This included collapsing themes into a hierarchical structure with up to three layers and further sub‐divisions between data from mothers and other groups (i.e., observers and the family and friends of mothers). Following each of the three meetings, AG updated and circulated the draft summary of the thematic analysis and an updated NVivo file where additional coding had been undertaken. At each stage, the research team reviewed these independently and discussed in the following meeting until the final themes were agreed by all researchers. Before publication, the searches were repeated from 2017 to 2021 by DM, and the additional papers were analysed using the existing coding framework. Following two additional data analysis meetings between AG, DW and AB, it was agreed that no new codes were required. This decision was agreed by email with all authors. Two authors (AG and RP) developed the figures displaying the results, which were agreed by all researchers.

## 3 RESULTS

### 3.1 Studies identified

**Figure 1:**
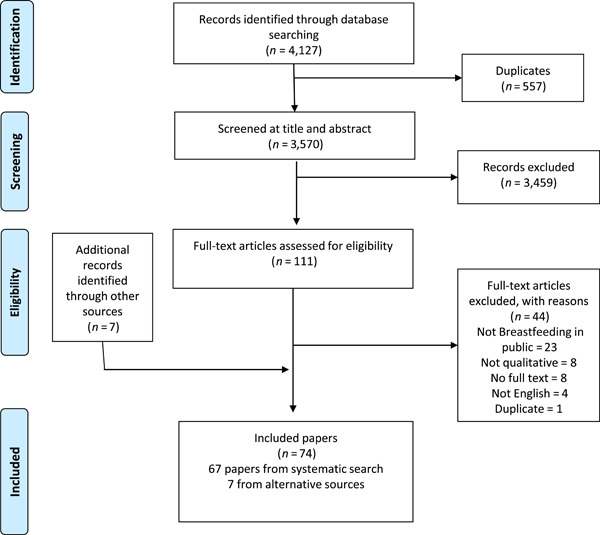
PRISMA flow chart of study selection

Database searching identified 3570 unique records, 111 of which were fully screened against the inclusion criteria. Additionally, 1235 books were screened for inclusion and the full text of 10 books or potentially relevant chapters were reviewed. One was included in the synthesis, with a second book identified through database searching. Unpicking systematic reviews, as well as forward and backward chaining identified additional seven eligible sources. The PRISMA flow diagram in Figure 1 illustrates this process (Moher et al., 2009).

### 3.2 Included studies

Seventy‐one studies were included in the review, which accounted for 74 papers as three studies had two papers published using the same data (Boyer, 2012, 2018; Bueno‐Gutierrez & Chantry, 2015; Chantry et al., 2008; Grant, 2015, 2016). The most common data sources used within the included studies were documents (i.e., a written or graphical artefact found in isolation from its author (Grant et al., 2019; Grant, 2022), although these were often small data extracts, such as tweets or comments within forums. With the exception of documents, data overwhelmingly came from pregnant women and mothers with over 17,700 participants divided mostly between survey and interview studies. Data collection between 1995 and 2020 were stated, although in 23 of the 71 cases the year of data collection was not stated. Interviews were the most utilised research method, although more than one research method was used in 15 of the studies. There was variation in approach between studies that employed interview methods, including online interviews and emails (Dowling & Pontin, 2017), visual methods (Grant et al., 2017) and repeat interviews (Helps & Barclay, 2015). A broad range of theoretical and analytical approaches was utilised. A summary of the participants, location, and data collection and analysis methods used within the included studies can be seen in Table 1. Characteristics of each individual study are summarised in Table 2.

**Table 1:** Overview of included studies

Characteristic	*N*
**Participants**	
Pregnant women and mothers	17,780
Male partners of pregnant women or mothers	156
Grandparents (all)	46
Grandmothers^a^	≥19
Family and friends of mothers	10
Health professionals and community Health champions	406
Employees in public facing businesses	15
Members of the public	438
Documents	15,449
**Location** b	
UK	22
USA	18
Australia	12
Canada	5
Ireland	5
Mexico	2
Sweden	2
Global (social media)	2
Finland	1
Italy	1
the Netherlands	1
New Zealand	1
Spain	1
Not reported (UK ethical approval)	1
**Data collection method** c	
Interviews	46
Focus groups	20
Surveys	8
Documentary analysis	7
Observations	4
Ethnography/autoethnography	2
Diaries	1
Not stated (appears ethnographic)	1

^a^ Grandmothers are also included in the combined grandparents figure.

^b^ NB: Two cases reported more than one location; Hauck et al. (2020) with three countries and Lehto (2019) with one country plus ‘global’ social media.

^c^ Many studies included more than one and up to three methods of data collection; all methods are included in this table.

**Table 2:** Characteristics of included studies

First author, year published	Setting					Study details					Quality	
	Year of data collection	Country, area	Breastfeeding rates (stated in paper)	Legal status of breastfeeding in public	Factors affecting results	Aim	Recruitment	Participants	Data collection	Data analysis	CASP (*indicates double screened)	Data quality
Alianmoghaddam, 2017	2013–2014	New Zealand, lower North Island	EBF 6M: 38%	Not stated	Intention to EBF for 6M Most: aged 25 years+; of European dissent (3 Māori, 3 immigrants); highly 3 immigrants); highly educated, married, full‐time employees with maternity leave of 6M+	Understand male partner support for breastfeeding in women who intended to EBF for 6M	Adverts in public places, breastfeeding social media websites and snowballing	30 heterosexual women who intended to EBF for 6M	Face‐to‐face postpartum interview Monthly telephone interviews to 6M	Thematic analysis	Yes: 9 Can't tell: 0 No: 1	High
Andrew, 2011	Not stated	UK, England, Reading	England: Initiation: 78% 6M: <1% EBF	Not stated	Affluent area; focus on BF	Consider BF decisions over first few months	Hospital, following birth	12 primi‐ and multi‐parous women who gave birth in Reading, Berkshire	Face‐to‐face interviews	Inductive code generation	Yes: 8 Can't tell: 2 No: 0	High
Avery, 2011	2002	USA, San Francisco, Chicago, New Orleans	Not stated	Not stated	Excluded participants with strong BF intentions	Understand views of BF to inform a public health campaign	Random digit dialling	81 pregnant women, 40 male partners of pregnant women. Half African American, half Caucasian	Focus groups	Constant comparative analysis approach	*Yes: 8 Can't tell: 1 No: 1	High
Battersby, 2007	Not stated	UK, North of England	Not stated	Not stated, but example of police asking women not to BF in public cited	Typically, lower BF rates than England as a whole	Not stated	Not stated	Interviews: 39 BF mothers; 10 midwives; a survey of 291 midwives	Interviews; survey (unclear if free text response options)	Not stated	Yes: 2 Can't tell: 6 No: 2	Low
Boyer, 2012 (A) & Boys, 2018 (C)	2007–2010	UK, Southampton Millbrook area	Unclear (UK or England): 6M: 25% any BF; <1% EBF	Not stated	Mothers mostly white, UK‐born, homeowners, in stable relationships	Consider how BF experience affects BF duration	Survey – NCT second‐hand clothing sale. Interviews – from parenting classes	Survey of 46 women; interviews with 9 mothers and 2 lactation consultants; 180 posts on a mothers' forum	Survey, interviews, and documentary analysis	Interpretivist framework; identifying cross‐cutting themes	Yes: 6 Can't tell: 4 No: 0	Medium
Boyer B	2009	UK, Southampton	UK; Initiation: 75% 6W: <50% EBF 6M: <1% EBF	Not protected by law outside of Scotland	Mothers defined as ‘middle class’	Consider BF in public and Lactation advocacy	Mothers group arising from a free parenting class in a deprived area	15 BF activists; 9 mothers – all white and heterosexual	Interviews; participant observation at BF picnics	Thematic analysis	*Yes: 3 Can't tell: 0 No: 7	Low
Brouwer, 2012	Not stated	Australia, region not stated. Southern suburbs of an Australian city	Not stated	Not stated	First‐time mothers	Investigate how social norms influence first‐time mothers' decisions around feeding method	Hospital at 1–7 days postpartum	11 healthy first‐time mothers with no birth complications; aged 21–41	Repeated semi‐structured interviews (2 interviews per participant)	Third‐level thematic analysis technique	Yes: 8 Can't tell: 0 No: 2	High
Brown, 2021	2020	UK	Not stated	Not stated	Data collected during COVID‐19 pandemic	Understand how the COVID‐19 pandemic affected infant feeding attitudes, choices and outcomes	Adverts on social media, shared by breastfeeding organisations	1219 mothers of infants aged <12 months who had breastfed at least once during the COVID‐19 pandemic	Online survey	Thematic analysis	Yes:9 Can't tell: 1 No: 0	High
Carlin, 2019	2016–2018	USA, Washington DC	USA: Initiation: 81% 6M: 52% any 12M: 31% any	Not stated	Mothers were African American or Caucasian. Focus groups stratified by race. Health professionals provided validation of analysis.	To understand perceptions and reactions to norms relating to BF	Participants in a larger quantitative study recruited via birth hospitals	28 mothers; 20 African American; 8 Caucasian,	Focus groups and in‐depth semi‐structured interviews	'Standard qualitative analytic'	Yes: 8 Can't tell: 2 No: 0	High
Cato, 2020	2017	Sweden, Uppsala County	1 week: 95% 1‐week EBF: 78% 6M EBF: 15%	Not stated	Participants aged 27–37 years. Most participants had a high‐level education	Explore attitudes to breastfeeding in pregnant women	Via parental classes at 3 maternity centres in urban and rural areas; also via midwife introductions and posters; Snowballing	11 pregnant women in late pregnancy	Semi‐structured interview	Thematic analysis	Yes: 10 Can't tell: 0 No: 0	High
Chantry et al., 2008	Not stated	Mexico, Tijuana (border town with San Diego)	North Mexico: <6M: 11% EBF Mexico: <6M 14%	Not stated, but appears that it is not protected as not mentioned in PhD thesis	Study area has a very low BF rate compared to Mexico as a country	Identify the main social obstacles to BF in a low‐income population in Tijuana, Mexico	Community health workers/waiting areas of health clinics	66 mothers; 11 fathers; 27 grandparents and 25 key informants	Focus groups, interviews, participant observation, document analysis	Thematic analysis using constant comparison	Yes: 9 Can't tell: 1 No: 0	High
Charlick, 2017	Not stated. Ethical approval in 2014.	Australia, region not stated	Australia: Initiation: 92% 6M: 18% EBF	Not stated	Only one participant: study recruitment materials suggest aimed to recruit more.	To understand what enabled a first‐time mother to continue exclusively BF between 2 and 6 months in Australia	Flyers in the community	1 first‐time heterosexual mother. 12 years' experience as a midwife.	Face‐to‐face interview (semi‐structured)	IPA	Yes: 7 Can't tell: 3 No: 0	High
Charlick, 2018	2015	Australia, (South)	Australia: Initiation: 92% EBF 6M: 18%	Not stated	Researcher is a midwife; interviews conducted in a health service centre	Explore reasons mothers who intended to EBF to 6M stopped EBF between 2 and 6 months	Community advertising flyer	5 new mothers who intended to EBF to 6M but EBF from 2 to 6 months	Semi‐structured interviews	IPA	Yes: 10 Can't tell: 0 No: 0	High
Charlick, 2019	Not stated	Australia	Australia: Initiation: 92% EBF 6M: 18%	Not stated	Respondents all married and Caucasian	Understand the experiences of women who intended to EBF to 6M and were successful in meeting their goal.	Community advertising flyer	5 new mothers who EBF to 6 months	Semi‐structured interviews	IPA	Yes: 9 Can't tell: 1 No: 0	High
Chiang, 2018	2016	USA, Texas	USA (all ethnicities) Introduction of solid food <4M: 40%	Not stated	Interviews in English but participants were bilingual and used Spanish phrases during interviews	Understand beliefs, motivation and behaviours of Hispanic WIC enroled mothers relating to mixed feeding (las dos) and early introduction of solid food	Invitational email to those identified as working with Hispanic WIC recipients. WIC Director's permission sought	15 WIC breastfeeding peer counsellors 2 regional breastfeeding coordinators	Key informant interviews Field notes	Miles and Huberman's 3 stage process	Yes: 6 Can't tell: 1 No: 3	Medium
Chopel, 2019	2015–2017	USA, Northern California	Not stated	Not stated	Young mothers; area with high poverty and gentrification; all study areas had high levels of young births; poor availability of health services. BF support in the areas not always viewed as accessible or welcoming	To describe social and structural barriers to BF in young mothers	Three areas: one Latino, one African American; one mixed. Service introduction, flyers, word of mouth	9 key informants 12 mother/decision‐making partner (6 dyads) 21 young mothers	Community‐Based Participatory Research. Interviews Dyad interviews Focus groups with mapping exercises	Grounded theory based, collaborative analysis including young mothers & IBCLCs	Yes: 10 Can't tell: 0 No:0	High
Condon, 2010	2009	UK, England, Bristol	UK: Initiation: 78% 6W: 50% any	Not stated	Evaluation conducted by volunteer BF supporters and health professionals (those delivering the intervention).	On the spot evaluation of an intervention to increase awareness of BF and change attitudes towards BF in public	Exhibition in 10 public spaces around Bristol (shopping centres, child health centres, walk‐in centre, health centre). Evaluation in 6 of these places.	158 participants – 71% female, 25% male. Aged from 10‐70. Most respondents aged 20 to 30.	Survey, including open text boxes	Not stated	Yes: 2 Can't tell: 3 No: 5	Low
Condon, 2018	2011–2012	UK, Southwest England	EBF 6M: <1%	Not stated	Migrant mothers BF more, but this decreases 5% for every 5 years in the UK.	Understand the experiences of parents born abroad who are raising children in the UK	Not stated	22 Migrant Roma mothers and grandmothers	Semi‐structured interviews	Not stated	Yes: 5 Can't tell: 3 No: 2	Medium
Dayton, 2019	2013–2015	USA, Midwest	Not stated	Not stated	Both parents had to be involved in the study High rates of poverty, violence, and mental illness.	Understand the worries, barriers, and promotive factors for BF in expectant mothers and fathers	Advertisements online and at social service agencies, obstetric clinics and community centres	95 third trimester mothers (48) and fathers (47) aged 18+ living in low‐income (46% below federal poverty line) impoverished urban environment	Semi‐structured interviews	Mixed methods Approach (involving quantitative data too) based on grounded theory	Yes: 9 Can't tell: 1 No: 0	High
DeMaria, 2020	2017	Italy, Florence	‘BF rates 86%’ (p1)	No law against BFP	Participants had to be proficient in conversational English. 37/44 had initiated or completed college. Most married/in a relationship	Explore women's (who were not mothers) perceptions, attitudes, and experience with breastfeeding	Social media adverts; flyers placed throughout city and handed out by researchers in public areas (libraries and cafes) & snowballing	44 women aged 18‐45 years who did not have children using the Italian health care system. Heterosexual (37%), Bisexual (6),	In‐depth interview in participants' choice of location using semi‐structured topic guide	Content analysis	Yes: 9 Can't tell: 1 No: 0	High
Dowling, 2017	2008–2009	UK, region not stated	UK: 6M: 34% any 6M: 1% EBF	Not stated	Mothers who breastfed for longer than 6 months (up to 4 years)	To use the concept of liminality to explore Experiences of women BF long term in the UK	Recruitment via La Leche League and other sources.	Observation: 70 mothers at BF Support groups Interviews: 10 mothers Range of hetero/homo sexual; single/in relationship; Age range 20s late 40s; some work/full‐time mum	Observation of BF groups (to see how women support each other). Online asynchronous interviews (OAI) via email. Face‐to‐face interviews	Thematic analysis	Yes: 8 Can't tell: 1 No: 1	High
Dyson, 2010	Not stated. Funding from 1999 to 2002.	UK, England: Leeds, Bradford, Birmingham, London	UK Initiation: 51%	Not stated	Deprived areas; aged 16–20; white ethnicity; low income; first‐time mothers	Explore psychosocial factors influencing infant feeding intention among pregnant teenagers expecting their first baby living in deprived urban areas of England.	Midwives (survey); staff of parenting education programme that was compulsory for benefit claimants (focus group)	Survey: 71 first‐time mothers Focus groups: 17 first‐time mothers. 15 had a partner. All low income, deprived area, teenage and white ethnicity.	Survey, focus groups	Framework analysis	*Yes: 8 Can't tell: 0 No: 2	High
Eni, 2014	Not stated	Canada: British Columbia, Manitoba, Ontario	Canada: Initiation: 87% 6M: 16% EBF	Not stated	First Nation women; birth/postnatal care takes place away from local community	Understand the experiences, strengths, and challenges of BF for First Nations women.	Not stated; purposive sampling used	52 mothers; 13 grandmothers 40% had graduated from high school; majority low/very low/low income	Focus groups using an Indigenous feminist standpoint	Qualitative methodological technique	Yes: 6 Can't tell: 4 No: 0	Medium
Foster, 2010	1999–2002	Australia, Melbourne	ABFAB trial participants, Australia: 6M: 3% EBF	Not stated	Enroled in a trial of breastfeeding education (not successful in promoting breastfeeding)	To explore women's views and experiences of BF, as part of the ABFAB trial (breastfeeding education)	6 month follow‐up of ABFAB trial	889 women who had taken part in ABFAB trial	Survey with 2 open questions	Simple thematic analysis	Yes: 3 Can't tell: 3 No: 4	Low
Furman, 2013	2009	USA, Cleveland	USA, Local inner‐city predominantly WIC eligible population 2M: 20% any	Not stated	African American women; ‘high risk’ (e.g.: domestic violence); inner‐ city; eligible for Moms First intervention; many WIC eligible	To identify barriers to BF among high‐risk inner‐city African‐ American mothers	Flyers and word of mouth	20 African American women eligible for Moms First intervention (pregnant or infant aged under 2 years)	Focus groups	Deductive analysis using Factors Influencing Beliefs model	Yes: 7 Can't tell: 1 No: 2	High
Gallegos, 2015	2007–2008	Australia, Brisbane & Perth	'Refugee women': an 8%–85% decrease in initiation and duration.	Not stated	Refugee women; in Africa EBF is not the norm and grandmothers strongly influence infant feeding	To explore the experience of BF among refugee women from Liberia, Sierra Leone, Burundi, and the Democratic Republic of Congo living in two major capital cities in Australia.	Women's community organisations and snowballing	30 refugee women and 1 man from Liberia, Sierra Leone, Burundi, and the Democratic Republic of Congo living in two major capital cities in Australia. Range of ages of children (2 months – 28 years) and duration in Australia (1 week	Interviews and focus groups	Thematic analysis	Yes: 9 Can't tell: 1 No: 0	High
Grant, 2016 (A); Grant, 2015 (B)	2014	UK	UK: 6M: 1% EBF	Protected by law	Data were from a single case study where a woman was stopped BF in a shop (her legal right) and mothers protested in the shop. This was reported by the Mail Online news site. Online disinhibition in comments	To examine on‐line opinion regarding BF in public and protesting about the right to breastfeed in public within the context of a single case.	Data were mined from the male online website and Twitter for 24 h after the article was posted. Data were captured using NCapture for Nvivo.	884 naturally occurring comments from Mail Online news site and 1210 tweets relating to a protest supporting women's right to breastfeed in public. Mostly appear to be ‘observers’.	Documentary analysis	(Grant, 2016); Critical Discourse Analysis (Grant, 2015); semiotic and thematic analysis	*Yes: 9 Can't tell: 0 No: 1	High
Grant, 2017	2014	UK, South Wales	UK: 6M: 1% EBF	Not stated	Deprived areas (Communities First) with high levels of health service intervention; necessity of grandmothers and mothers being in regular contact	To understand infant feeding experiences and decision‐making in mother/grandmother dyads from deprived areas	Researcher contacts and snowballing	6 mother/grandmother dyads. All white. Mothers had infants aged under 30 months.	Intergenerational dyad artefact elicitation interviews	Inductive thematic analysis	*Yes: 8 Can't tell: 0 No: 2	High
Grant, 2019	2016	UK, South Wales	Not stated	Not stated	Non‐participants sometimes present during interviews. Three interviews per person, using creative tasks to guide an elicitation interview	Use creative methods to facilitate discussion of views and experiences of health behaviour and pregnancy	External to health service. Snowballing + social media	10 Pregnant women (<30 weeks gestation) living in highest quintile of deprivation and claiming means tested welfare benefits	Creative methods– pre‐interview tasks followed by elicitation interviews) (timelines, collaging, ‘thought bubbles’ dyad sandboxing); three interviews per person. Field diaries	Thematic analysis (deductive and inductive) and mapping to COM‐B model	Yes: 10 Can't tell: 0 No: 0	High
Grant, 2021	2018	UK, Cardiff	Not stated	Not stated	Locations under study identified from previous literature on BFP. Researcher was not a mother	Investigate social‐spatial aspects of public spaces in one UK city to suggest barriers and facilitators to BFP	n/a	Locations: several areas of the city, transport, transport hubs, high streets, cafes and shopping centres	Urban ethnography Low inference field notes	Thematic analysis (deductive and inductive)	Yes 8 Can't tell: 2 No: 0	High
Hauck, 2020	2018	Australia, Ireland, Sweden	Initiation: Australia: 92% Ireland: 55% Sweden: 98% 6M: Australia: 60% Ireland: 26‐29% Sweden: 72%	Legally protected in all three countries	Three open text questions were asked. Participants mostly had a high level of education	To explore what women from three high‐income countries perceived as helpful or challenging when breastfeeding in public.	Social media. Survey open for four weeks in each country	Women who were breastfeeding or had breastfed in the past 2 years 10,910 from Australia 1835 from Ireland 1520 from Sweden	Cross‐sectional online survey	Content analysis	Yes: 9 Can't tell: 1 No: 0	High
Helps, 2015	Not stated	Australia, New South Wales	Australia: Initiation: 96% 4M: 39% EBF 6M: 15% (unclear if any or EBF)	Not stated	Aboriginal women (colonialism impacts on infant feeding); several participants from deprived areas.	To explore the factors impacting upon infant feeding choices in a rural Aboriginal Community.	Appointed Aboriginal maternity care workers recruited patients	8 Aboriginal mothers; 5 Aboriginal health workers, 2 Aboriginal BF champions	Semi‐structured repeat (ante‐natal and post‐natal) interviews using Indigenist methodology	Inductive thematic analysis	Yes: 9 Can't tell: 1 No: 0	High
Henderson, 2011	1999–2000	UK, Leeds (England) & Glasgow Scotland	Leeds: 6W: 48% any; 37% EBF	Not stated	Deprived areas and low income	To understand the views of father in relation to BF and formula feeding	Unclear	28 low‐income men in areas of deprivation, aged 16‐45. Range of parents, expecting or neither.	Focus groups	‘some of the principles of grounded theory’	*Yes: 8 Can't tell: 1 No: 1	High
Hinson, 2018	2016	USA, North‐Eastern city	Initiation USA: 81% USA African American: 66%	Not stated	African American women High proportion living in poverty (Medicaid eligible)	To uncover influences on opinion, behaviour and motivation for BF and explore social issues affecting African American women initiating BF.	Primary care facility with high % African American patients. 75% eligible for Medicaid. Recruitment by nursing/medical staff	34 African American mothers of healthy‐term babies aged 0‐ 3 months.	Focus groups (*n* = 6)	Conventional qualitative Content analysis	Yes: 8 Can't tell: 2 No: 0	High
Isherwood et al., 2019	2017	UK, Bristol	Bristol: Initiation: 81% 6‐8 weeks: High income area: 85% Low Income area: 30%	Legally protected	Bristol England's first BF‐friendly city. The areas were chosen to have the highest and lowest rates of breastfeeding in the city Mothers who succeeded at BFP	Explore breastfeeding experiences in two neighbourhoods of the city	Local breastfeeding support groups; a local Facebook breastfeeding group; snowballing	22 mothers – 11 high‐income area, 11 low‐income area	Semi‐structured interviews	Thematic analysis	Yes: 9 Can't tell: 1 No: 0	High
Jamie, 2020	Not stated (ethics in 2014 and 2016	UK, Belfast, Bristol, Middlesbrough,	UK: Initiation: 81% EBF 1 week: 46% EBF 3M: 17% EBF 6M: 1%	Not stated	Young mothers; deprived area	Study 1: To examine adolescent mothers' health beliefs and behaviours Study 2: to refine and expand insights relating to BF	Through children's centres and organisations providing services to young parents	27 adolescent mothers (<21 years at birth of first child) from deprived areas) 5 children's centres staff	Study 1: Photo elicitation (mums' photos) repeated focus groups; Study 2: follow‐up interviews with 3 mothers; Health professional interviews	Constant case comparison and deviant case analysis	Yes: 10 Can't tell: 0 No: 0	High
Lee, 2019	2016	UK, Bath	Not stated	Not stated	All = professional mothers, on maternity leave, intending to return to work	To understand first‐time mothers' experiences of loneliness	ViaMumsnet.com/bath and bathmums.com	7 first‐time mothers (babies aged 4‐9 months)	Semi‐structured interviews	IPA	Yes: 9 Can't tell: 1 No: 0	High
Leahy‐Warren, 2017	Not stated: ethical approval in 2013	Ireland, region not stated	Ireland: 4W: 60% any 6M: very low (only 1 participant in the study giving any breastmilk)	Not stated	Attendees of BF support group. Most women were primiparous, married and had a vaginal delivery.	To explore BF women's experiences of a Public Health Nurse‐led support group	Leaflets given out at BF support group or groups. Asked to register interest.	7 women who had used a BF support service	One‐to‐one semi‐structured face‐to‐face interviews	Thematic content analysis	Yes: 8 Can't tell: 1 No: 1	High
Leeming, 2013	2006–2007	UK, England, Midlands	UK: Initiation: 4/5 6W: <50% any 6M: <1% EBF	Not stated	First‐time mothers. All reported those that they lived with were supportive of BF. Majority were higher socio‐ economic status. High level of dropout between phases 1 and 2.	To explore BF first‐time mothers' perspectives of the social context of their infant feeding	Advertised in GP surgeries and antenatal clinics. Asked to register interest.	22 first‐time mothers, all lived with a male partner. 2 of the mothers were teenagers living with other family members too.	Audio diaries for 7 days starting 1‐3 days after birth, and elicitation interview. Repeated 3‐4 weeks later (*n* = 11 diaries, 13 interviews)	Thematic analysis	Yes: 9 Can't tell: 1 No: 0	High
Lehto, 2019	2018	Finland/global (social media)	Not stated	n/a	Related to a single case study; collected 2 years after the event – content may have been deleted. Excludes private accounts.	Not stated	n/a	Content responding to one Instagram post of a mother BF on an aeroplane and responses using the hashtag #teriniitti – mostly from Jan 2016	Documentary analysis: social media posts (including images) (2813); media articles (5); press statements (1); online forum (370)	Content analysis & thematic analysis	Yes: 4 Can't tell: 5 No: 0 Not applicable: 1	Low
Llorente‐Pulido, 2021	2018–2020	Spain, Canary Islands, Tenerife	Spain: 6w: 81% 3M: 76% 6M: 58% EBF 6W: 66% EBF 3M: 53%	Not stated	High unemployment rate in Tenerife; population widely dispersed impacting health services; most of population live in urban areas	Understand midwife's perspective on bio‐psycho‐social factors that undermine EBF	Snowball including the use of an initial key informant responsible for BF groups on the island	20 (of 53) midwives working in primary care centres in Tenerife, working in both urban and rural areas – heterogeneous sample	In‐depth interviews	Content analysis	Yes: 9 Can't tell: 1 No: 0	High
Majee, 2017	2015	USA, Midwest	Not stated	Not stated	All parents were cohabiting and heterosexual. Majority married	Understand how co‐parents collaborate around infant and toddler feeding	Paediatric clinic flyers	24 mother/father dyads (infants aged 6–36 months)	Dyad semi‐structured interviews	Thematic analysis	Yes: 9 Can't tell: 1 No: 0	High
Marcon, 2018	2017	Global: Instagram	n/a: global	n/a: global	700 million users; more common with women than men, and younger people than older people	Determine if and how BF is promoted and supported on Instagram	Popular BF‐related hashtags: #breastfeeding, #breastmilk, #breastisbest, and #normalise breastfeeding	4089 images 8331 comments related to popular BF hashtags. Videos and duplicates excluded	Instagram's Application Programming Interface (API)	Content analysis	Yes: 8 Can't tell: 1 No: 0 Not applicable: 1	High
Marsden, 2012	2009	Not stated. Appears to be UK. Ethical approval from Liverpool John Moores University and ‘local businesses’ were	UK: Initiation: 81% 6W: 48% any 6M: 25% any	Protected by law	One area; staff recruited from 5 businesses.	To explore attitudes and opinions towards breastfeeding in public among employees working in public spaces	Information sheet and consent form sent by post to selected businesses.	9 employees from public places with (4) and without (5) Breastfeeding facilities/policies 3 were parents with breastfeeding experience.	Semi‐structured interviews	Thematic analysis (inductive and deductive)	Yes: 7 Can't tell: 1 No: 2	High
Mathews, 2019	2013–2017	Canada, Regina	Canada Initiation: 89% EBF 6M: 26%	Legally protected	Situated within feminist autoethnography	Examine the embodied practice of BF in urban public spaces	n/a	One researcher: white, middle class, heterosexual, highly educated, in her 30s	Autoethnographic vignettes	Not stated	Yes: 6 Can't tell: 1 No: 0 Not applicable: 3	Medium
McKenzie, 2018	2013–2014	USA, New York	Hospitals participants recruited from: Initiation: 84%–95%	Not stated	BF in public may be more difficult for obese women due to more breast tissue. Only one of four hospitals recruited from was BFI accredited	Describe experiences of BF in public and compare experiences of obese and non‐obese women	Flyers in hospital; snowballing	26 pregnant women in third trimester intending to breastfeed who gave birth to a healthy baby; normal weight or obese	Longitudinal (minimum of 2, maximum of 5) semi‐structured interviews	Conventional qualitative analysis	Yes: 10 Can't tell: 0 No: 0	High
Morris, 2016	2015	UK	UK: 6W: 23% EBF 6M: 1% EBF	Protected by law	Focus on single incident of women asked to leave expensive restaurant, Claridges. Online disinhibition.	To assess abuse of breastfeeding in public in the UK and understand why some UK residents object this practice	Online search for ‘Claridges’ and ‘breastfeeding’	805 comments from 12 news media websites and parenting forums	Documentary analysis	Thematic analysis	Yes: 7 Can't tell: 0 No: 2 Not applicable: 1	High
Newman, 2018	Not stated	England, East	England 6–8 weeks: 44%	Legally protected	Mid‐sized market town; Mothers all white, heterosexual, in a long‐term cohabiting relationship	Provide insight into mothers BF longer term in an area where it is nonnormative	Poster in three children's centres in town and local area Facebook breastfeeding groups	12 Mothers feeding infant over 6M of age	Semi‐structured interviews	IPA	Yes: 9 Can't tell: 1 No: 0	High
Nesbit, 2012	2008–2009	Canada, Ontario, Durham region	Canada: Initiation: 95% Adolescent initiation (<19 years): 84% Durham region: Adolescent initiation (aged 15‐19 years): 79%	Not stated	Young mothers, did not recruit in rural areas, first‐time mothers.	To examine barriers and facilitators to breastfeeding in adolescent mothers in one region of Ontario, Canada	Posters in health and social care agencies. Asked to register interest.	16 adolescent mothers (15–19 years) with infants aged less than 12 months who had BF at least once	Face‐to‐face semi‐structured interviews	Conventional content analysis	*Yes: 10 Can't tell: 0 No: 0	High
O'Sullivan, 2021	2015–2016	Ireland	Ireland Hospital discharge: Any: 60% EBF: 50%	Not stated	Immigrant status	Describe BF experiences and attitudes among Polish mothers living in Ireland	Notices in Polish churches and schools, preschools, and a parenting social network. Snowballing via key informants.	16 Polish mothers of term birth babies within the last year who had lived in Ireland for 10 years or less	Semi‐structured interviews	Qualitative thematic analysis	Yes: 9 Can't tell: 1 No: 0	High
Owens, 2018	Not stated	USA, Central Florida	Not stated	Not protected in some states of the USA	African American women, recruited externally to WIC	To contribute to the experiential literature on African‐ American mothers and breastfeeding in public.	Recruited through health care providers and snowballing	22 African American mothers, aged >18 years, with infants younger than 1 year. All BF at the time of interview. 64% married.	In‐depth semi‐structured interviews	Constructivist grounded theory	Yes: 8 Can't tell: 0 No: 2	High
Pallotti, 2016	2013–2014	UK, England, Sheffield	UK: Initiation of those who left school aged 16: 63%	Not stated	Young mothers with an interest in breastfeeding	To explore the lived experiences of 10 mums aged 16–18 using interviews and participant observation	Recruited through teenage pregnancy midwives	10 pregnant women who were ‘reasonably well’ aged 16–18 with an interest in breastfeeding	Ethnographic interviewing, non‐participant observation from pregnancy to weaning	Thematic network analysis	Yes: 10 Can't tell: 0 No: 0	High
Prendergast, 2016	Not stated	Australia (location withheld for confidentiality)	Australia Initiation: 96% EBF 3M: 39% EBF 5M: 15%	Not stated	Using personal reflective statements which were produced for the purpose of gaining an educational qualification in BF	Pilot study to inform the development of a larger study to determine women's experiences of BF	Via Australian BF Association education administrator	20 Australian BF Association trainee counsellors, taking Certificate IV in BF education	Documentary analysis of personal BF experiences reflective statement’	Thematic analysis	Yes: 8 Can't tell: 2 No: 0	High
Rhoden, 2016	Not stated	USA, Washington DC/Maryland	USA Black (African American women: Initiation: 30%	Not stated	African American men; mostly associated with a faith‐based organisation. Narrow topic guide	What are the sociocultural factors that influence African American men's perceptions of breastfeeding.	Recruited through faith‐based organisations and non‐profit organisations	17 African American men aged 18 plus	Focus groups	Data were mapped to the socio‐ecological model	Yes: 6 Can't tell: 0 No: 4	Medium/Low
Robinson, 2011	Not stated	USA, Mid‐West two cities with high numbers of African American women seeking care	USA: Initiation: 75% African American Initiation: 60% 6M: 28% any 12M: 13% any	Unclear, but ‘more work is necessary to change laws…’ (p. 327)	African American women. Many were multi‐parous. Likely the majority were living on a low income.	To examine prenatal breastfeeding self‐efficacy and infant feeding decisions among African American women using a black feminist philosophy situated in a mixed‐methods approach.	Patients of two health care centres were invited to fill in a survey. 59 did, 17 were invited to interview.	17 African American women. Mix of antenatal feeding intention. Low income likely. 3–4 weeks after birth	Narrative interviews situated within Black feminist philosophy	Narratives considered for Bandura's sources of self‐efficacy and themes developed	Yes: 9 Can't tell: 0 No: 1	High
Robinson, 2019	2017	USA (whole country)	USA African American women Initiate: 69% EBF 6M: 17% 12M: 24%	Not stated	Use of Black Feminist Thought throughout the research design may have increased rapport. Focus groups used to shift power dynamic from researchers to historically marginalised group	Describe the experiences of first‐time African American mothers who use breastfeeding Facebook groups	6 of the 9 Facebook BF groups for Black women allowed recruitment; flyers also on general Facebook and Instagram BF pages	22 African American women	Online focus groups situated within Black Feminist Thought	Thematic analysis	Yes: 10 Can't tell: 0 No: 0	High
Rose, 2012	Not stated	USA, region not stated for anonymity	Not stated	Not illegal, but women are sometimes treated as though it is (escorted out of premises by police).	The lactation room is based on a University campus.	To use a Foucauldian lens to explore the ways in which a lactation room functions as heterotopian space	Not stated	A single lactation room, on an American university campus, is described alongside comments from lactation room users, media coverage and author	Not stated, appears ethnographic/auto‐ ethnographic	Unclear. Abstract references rhetorical analysis	Yes: 3 Can't tell: 6 No: 0 Not applicable: 1	Low
Schafer, 2018	2015	USA, Iowa	USA: Initiate: 81% 12 months: 11%	Not stated	USA Identified as last of 36 developed countries in terms of support for BF. Restricted age range to 18–35. Mixture of university staff/students and WIC clinic users	Describe first‐time mothers BF experience from initiation to cessation and identify ‘turning points’ in BF journeys	Convenience sample: Mass email at a large university; flyers; in‐person recruitment at WIC clinics	28 first‐time mothers	Semi‐structured interviews	Thematic analysis	Yes: 9 Can't tell: 1 No: 0	High
Schindler‐Ruwisch, 2019	Not stated	USA, Washington DC	EBF 6M African American women: 17% African American in Washington DC: Initiation: 57% 6M: 26%	Not stated	Sample from two wards where BF rate for WIC recipients was the lowest	To learn about infant feeding practices and decisions	Recruited at four WIC clinics within two wards of Washington DC	24 WIC recipients	Semi‐structured interviews	Inductive and deductive coding	Yes: 10 Can't tell: 0 No: 0	High
Schmied, 2019	Not stated	Australia, Melbourne and Sydney	Australia Initiation: 96% 4M: 69% EBF 3M: 39%	Legally supported	Both study sites had one of the lowest BF initiation rates in their states	Identify community items that promote and support breastfeeding and early parenting	Staff from two local councils recruited via letter, email. Post or telephone. Reminders given	35 members of the community 6 business owners/managers	Appreciative Inquiry/community conversation workshop Focus group (business owners)	Qualitative content analysis	Yes: 9 Can't tell: 1 No: 0	High
Sheehan, 2019	Not stated	Australia, Sydney	Australia Initiation: 96% EBF 5M: 15% 6M: 60%	Protected	Disadvantaged area; most women born in Australia; mixed education levels	Explore perceptions and beliefs re: BF in public held by first‐time expectant mothers and their families	Not stated: part of a larger study	50 individuals from 9 families	Family conversations	Descriptive contextual analysis	Yes: 8 Can't tell: 2 No: 0	High
Shortt, 2013	Not stated	Ireland, Dublin	Ireland: Initiation: 55% Dublin: 4W: 20% EBF	Not stated	Low‐income mothers; mothers of infants up to 5 years (recall bias)	To explore infant feeding decisions among low‐income women living in Ireland.	Recruitment via staff of two community programmes and three primary healthcare centres	33 low‐income (receipt of benefit or living in deprived area) mothers with children aged under 5 years from urban and rural areas. All Caucasian; all but one was a native English speaker	Focus groups semi‐structured interviews	Inductive thematic analysis	Yes: 9 Can't tell: 0 No: 1	High
Spurles, 2011	2008	Canada, New Brunswick and Nova Scotia, Tantramar region	Rates in Eastern Canada are lower than in Western Canada	Not stated	All participants known to at least one moderator	To explore attitudes held by university educated young men and women residing in New Brunswick and Nova Scotia, Canada, about breastfeeding in public places.	Convenience sample recruited by research assistants/moderators using personal contacts	20 women and 27 men aged between 18 and 23. The majority were university students (22 men, 16 women); the rest had completed a university degree. 46/47 normally lived in Canada or the USA. All participants wanted their future children to be breastfed	Focus groups (single sex). Mixture of questions and photo elicitation	Narrative analysis	Yes: 6 Can't tell: 1 No: 3	Medium
Stav, 2019	2015	Netherlands, Nijmegen‐ Arnhem region	EBF 6M: 40%	Not stated	Mostly highly educated	To understand physical aspects of the environment that promote BF.	Personal and professional contacts	8 women currently breastfeeding who were secular and liberal in viewpoint NB: also draws on data from 4 women interviewed	Photo elicitation (researchers' photos) interviews	Not stated	Yes: 7 Can't tell: 3 No: 0	High
Stearns, 2011	1995–1998; 2005–2006	USA, Northern California	USA: Initiation: 74% 12M: 20%	Protected in ‘many states’	Majority of participants had breastfed an infant for 6 months plus. Large, class diverse sample, but mostly white/heterosexual.	To analyse in‐depth interviews with 66 breastfeeding mothers in Northern California in relation to extended breastfeeding	Snowball from a range of organisations including WIC, mothers, and a teen parenting club.	66 women. Aged 18–42 years. Class diverse. 82% of the sample identified as white, 11% as Latina, and 6% as American Indian/Native American. Two lesbians, 3 single mothers, 61 heterosexual couples. 88% currently breastfeeding; 79% had breastfed at least one child for 6 months	In‐depth interviews	An inductive approach	Yes: 7 Can't tell: 2 No: 1	High
Stevenson, 2019	Not stated but likely 2017	Australia, Corangamite and Moyne Shires	Not stated	Legally protected	Not all mothers had noticed the BF Welcome Here stickers (only 65% had)	An evaluation of the Australian BF Association's Breastfeeding Welcome Here intervention	Not stated	(3rd survey only) 23 mothers	Online survey	Not stated	Yes: 6 Can't tell: 4 No: 0	Medium
Swigart, 2017	2013	Mexico, central and southern	EBF 6M: 14% (Mexico) EBF 6M: 19% (rural & Indigenous areas of Mexico)	Not stated	Parents in receipt of means tested benefits. Rural v urban areas (known higher rates in rural areas)	Understand intention, practices, views in low‐resource communities	Snowball through community leaders and local health centres. Mothers of infants <2 years	10 fathers 50 Mothers – Prospero (poverty alleviating benefit) beneficiaries. 44 Community leaders Rural locations 60–80 min from state capitals; urban locations in capital	Interviews (fathers); Focus groups (mothers; community leaders)	Thematic analysis (deductive linked to theory of planned behaviour)	Yes: 8 Can't tell: 0 No: 0 Not applicable: 2	High
Thomson, 2015	2008–2010	UK, Northwest England	UK: 1W: 46% EBF 6M: <1% EBF	Not stated	Sample mirrored local breastfeeding rates. High rate of white and married or living with partner.	Secondary analysis of an evaluation of UNICEF UK baby‐friendly initiative award in two areas describing how discourses of shame are evident within the experience of breastfeeding and non‐breastfeeding women	Health professionals and coordinators of mother and baby groups or clinics asked mothers to participate and passed on contact details	63 women with some experience of breastfeeding; 43 were breastfeeding at the time of interview. Range of deprivation statuses. 59 were white; 62 were married/living with partner.	Focus groups semi‐structured interviews	Framework analysis drawing on Lazare's categories of shame	Yes: 7 Can't tell: 2 No: 1	High
Ware, 2014	2011	USA, Tennessee, Memphis and Shelby County	Tennessee: Initiation: 59% 6M: 4% EBF Tennessee African American: Initiation: 45%	Protected by law (Tennessee)	Use of incentives encourages a wider demographic to attend. Majority of participants were African American. Recruitment from areas with low breastfeeding rates.	To explore low breastfeeding rates in southeastern United States among African‐ American women using focus groups to identify perceived barriers	Recruitment flyers in a range of community organisations in areas with low breastfeeding rates, stating an incentive was available.	86 participants. Women of childbearing years (*n* = ?), men (*n* = ?), mothers (*n* = 7), and teens (*n* = 16). 40 of the participants were aged between 20 and 29, all bar two were African‐ American. Most participants were native to Memphis.	Focus groups	The Long Table Approach	*Yes: 8 Can't tell: 0 No: 2	High
West, 2017	Not stated	Canada, Nova Scotia	Canada: 4M: 50% EBF 6M: 26% EBF	Not stated	Students from one university. Relatively short interview duration (around 30 min) may indicate lack of rapport.	To explore the BF experience of students on the university campus in Canada	Campus‐wide email invitation, class presentations and snowball sampling	8 women who were current students (6), or alumni (2) in the past five years who have breastfed or intended to breastfeed an infant younger than one year whilst a student	Semi‐structured interviews	Qualitative thematic analysis built on Bandura's social cognitive theory	Yes: 8 Can't tell: 0 No: 2	High
Zhou, 2020	2009–2010	Ireland	EBF 6M 15% (Ireland); EBF 6M 21% (China)	Not stated	Immigrant mothers; BFP attitude positive in China (p8). Interviews conducted in Mandarin & translated	Understand successful EBF in Chinese immigrant mothers in Ireland	Purposive from respondents to Ireland Chinese Mother Survey	14 mothers Born in China, lived in Ireland for >6M, gave birth in Ireland, EBF for 4–6M	Semi‐structured face‐to face interviews. Field notes	Content analysis	Yes: 9 Can't tell: 1 No: 0	High

Abbreviations: BF, breastfeeding; EBF, exclusive breastfeeding; M, month(s); W, weeks.

### 3.3 Quality of included studies

The majority of papers were high quality according to CASP (Table 2) with few medium (*n* = 7) or low (*n* = 6) papers. However, we felt that the CASP scoring system was not always indicative of research quality. For example, Rose (2012) contained rich and interesting data but, due to limited methodological content, scored ‘low’. Opposingly, Rhoden (2016) was a doctoral dissertation which appeared to be poorly executed in several aspects but was rated as ‘medium’ quality based on its CASP score.

### 3.4 Thematic synthesis

**Figure 2:**
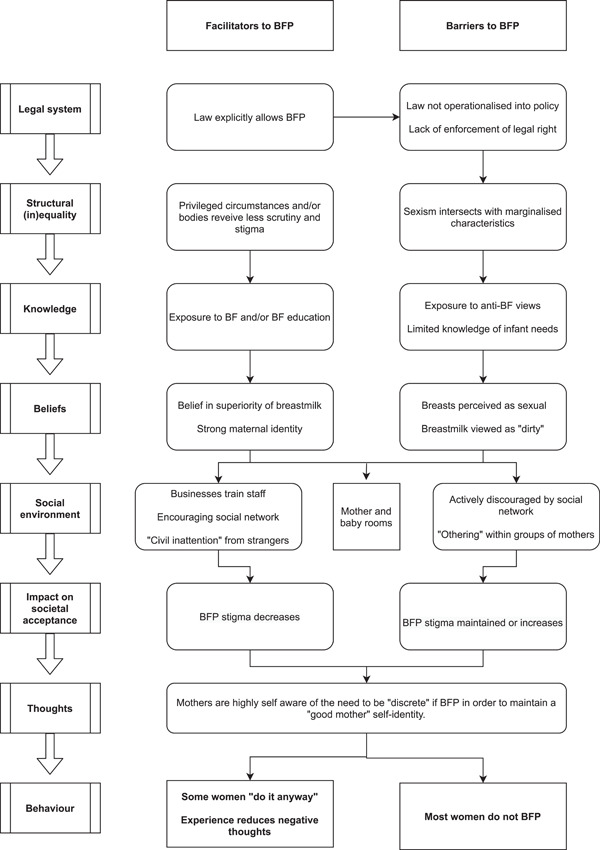
Barriers and facilitators to breastfeeding in public spaces: A thematic synthesis

Within the synthesis, the *high‐level societal contexts* were inferred from small extracts of data found in multiple included studies, which were combined to develop a new theme as per the qualitative synthesis guidance (Thomas & Harden, 2008). This inference was based on a range of explicit lower‐level societal discourses extracted from the data. Findings relating to knowledge, beliefs, space and the interactions between mothers and members of the public are reported based on the data within the included papers, with little inference required. Table 3 provides a summary of themes within individual articles, and a graphical representation of our thematic synthesis can be found in Figure 2.

**Table 3:** Summary of themes presented by study

Study			Societal barriers and facilitators to breastfeeding in public										Mothers' response to societal barriers and facilitators				
			Legal system		Structural inequality		Knowledge		Beliefs		Social environment		Mothers' thoughts		Mothers' behaviours		
First author	Date	Country	Facilitators	Barriers	Powerful group	Marginalised group	Facilitators	Barriers	Facilitators	Barriers	Facilitators	Barriers	Pro‐BFP	Negative BFP	BFP no issues	BFP ‘doing it anyway’	Don't BFP
Alianmoghaddam	2017	New Zealand									✓		✓	✓	✓		✓
Andrew	2011	UK			✓			✓		✓		✓		✓	✓		✓
Avery	2011	USA				✓		✓		✓		✓		✓			
Battersby	2007	UK			✓		✓	✓	✓	✓	✓	✓	✓	✓	✓	✓	✓
Boyer	2012 and 2018	UK	✓							✓	✓	✓	✓	✓	✓	✓	✓
Boyer	2009	UK			✓	✓	✓		✓	✓		✓	✓	✓		✓	✓
Brouwer	2012	Unknown								✓	✓	✓		✓		✓	✓
Brown	2021	UK												✓			✓
Carlin	2019	USA								✓		✓		✓		✓	
Cato	2020	Sweden						✓		✓				✓			
Chantry	2006	Mexico						✓		✓		✓		✓		✓	
Charlick	2017	Australia								✓	✓	✓	✓	✓		✓	✓
Charlick	2018	Australia				✓		✓		✓	✓	✓	✓	✓		✓	✓
Charlick	2019	Australia											✓	✓		✓	
Chiang	2017	USA				✓		✓				✓		✓			✓
Chopel	2019	USA				✓	✓				✓	✓		✓		✓	✓
Condon	2010	UK					✓	✓	✓	✓	✓						
Condon	2018	UK				✓			✓	✓	✓	✓	✓	✓	✓	✓	✓
Dayton	2019	USA						✓		✓		✓					
DeMaria	2020	Italy					✓	✓		✓	✓	✓				✓	
Dowling	2017	UK				✓			✓	✓	✓	✓	✓	✓	✓	✓	✓
Dyson	2010	UK				✓				✓		✓		✓			✓
Eni	2014	Canada				✓	✓	✓		✓		✓	✓	✓		✓	✓
Forster	2010	Australia								✓		✓	✓	✓	✓	✓	✓
Furman	2013	USA				✓		✓		✓		✓		✓		✓	✓
Gallegos	2015	Australia				✓	✓			✓		✓	✓	✓		✓	✓
Grant	2016 and 2015	UK	✓	✓			✓	✓	✓	✓		✓		✓		✓	✓
Grant	2017	UK				✓	✓	✓		✓		✓	✓	✓		✓	✓
Grant	2019	UK										✓		✓			
Grant	2021	UK									✓	✓					
Hauck	2020	Australia	✓			✓	✓		✓	✓	✓	✓		✓		✓	
Helps	2015					✓		✓		✓		✓	✓	✓	✓	✓	✓
Henderson	2011	UK				✓	✓	✓	✓	✓	✓	✓					
Hinson	2018	USA										✓		✓		✓	
Isherwood	2019	UK	✓		✓		✓				✓	✓	✓	✓		✓	
Jamie	2020	UK						✓		✓	✓	✓		✓			
Lee	2019	UK												✓			
Leahy‐Warren	2017	Ireland			✓					✓		✓		✓		✓	✓
Leeming	2013	UK		✓	✓					✓	✓	✓		✓		✓	✓
Lehto	2019	Finland							✓	✓	✓	✓					
Llorente Pulido	2021	Spain								✓		✓					
Majee	2017	USA									✓	✓		✓			
Marcon	2019	Global	✓				✓	✓	✓	✓	✓	✓	✓	✓		✓	
Marsden	2012	UK					✓	✓	✓	✓	✓	✓	✓	✓		✓	
Mathews	2018	Canada			✓	✓				✓	✓	✓		✓		✓	✓
McKenzie	2018	USA				✓						✓	✓	✓		✓	✓
Morris	2016	UK		✓				✓	✓	✓		✓		✓			
Newman	2018	UK				✓				✓	✓	✓				✓	
Nesbitt	2012	Canada				✓				✓		✓		✓		✓	✓
O'Sullivan	2021	Ireland									✓	✓		✓		✓	
Owens	2018	USA	✓	✓		✓		✓		✓		✓	✓	✓		✓	✓
Pallotti	2016	UK		✓		✓		✓	✓	✓	✓	✓	✓	✓		✓	✓
Prendergast	2016	Australia				✓	✓	✓			✓	✓	✓	✓		✓	
Rhoden	2016	USA				✓		✓		✓		✓					
Robinson	2011	USA				✓				✓		✓		✓		✓	✓
Robinson	2019	USA							✓		✓		✓	✓	✓	✓	
Rose	2012	USA				✓				✓		✓					✓
Schafer	2019	USA									✓					✓	
Shindler‐Ruwisch	2019	USA								✓	✓					✓	
Schmeid	2019	Australia					✓				✓	✓				✓	
Sheehan	2019	Australia	✓							✓	✓	✓	✓	✓		✓	✓
Shortt	2013	Ireland				✓				✓		✓		✓		✓	✓
Spurles	2011	Canada	✓				✓	✓	✓	✓		✓					
Stav	2019	Netherlands								✓	✓	✓		✓		✓	
Stearns	2011	USA				✓	✓	✓		✓		✓		✓		✓	✓
Stevenson	2019	Australia	✓				✓				✓			✓	✓	✓	
Swigart	2017	Mexico				✓	✓			✓	✓	✓		✓		✓	
Thomson	2015	UK								✓		✓		✓		✓	✓
Ware	2014	USA		✓		✓		✓		✓		✓		✓		✓	✓
West	2017	Canada				✓		✓		✓		✓		✓			✓
Zhou	2020	Ireland									✓	✓				✓	✓

#### 3.4.1 High‐level social contexts

**Figure 3:**
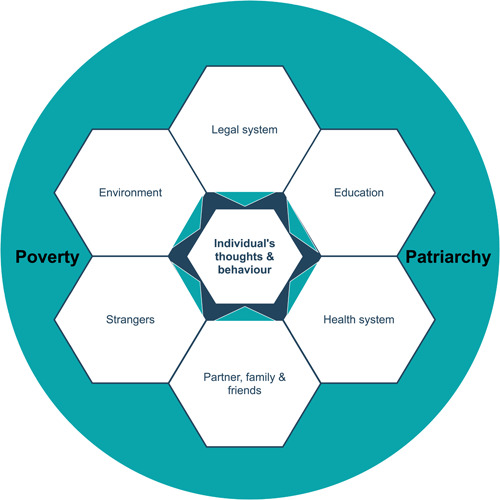
Concepts impacting on mothers' breastfeeding in public thoughts and behaviour

Our inferred social contexts focused on two major issues that were beyond the control of the individual citizen: legal protection for breastfeeding in public spaces, and intersectional inequality, which we framed through a lens of patriarchal misogyny (such as racism) and its associated impact on individuals who were more closely observed, and sometimes stigmatised by, authority figures and members of the public (Figure 3).

### 3.5 Legal system

We inferred from the data that legal systems were generally not actively supportive of breastfeeding in public, either due to authors not reporting the legal context (see Table 2) or stating that the law was poorly enforced. We also noted a relative lack of discussion of the legal context by mothers (see Table 3), although in seven papers mothers' knowledge of legal protections for breastfeeding in public provided maternal confidence (Hauck et al., 2020; Isherwood et al., 2019; Marcon et al., 2019; Owens et al., 2018; Sheehan et al., 2019; Spurles & Babineau, 2011; Stevenson, 2019). Some mothers also noted that they were prepared to strongly assert their *right* to breastfeed in public if confronted by a stranger, although this was not explicitly linked to stating their *legal* right or knowledge of it (Boyer, 2011, 2012; Charlick et al., 2017; Grant et al., 2017; Pallotti, 2016). However, other mothers expected their right to breastfeed in public to be ignored or actively challenged (Owens et al., 2018).

Where papers' authors reported that breastfeeding in public was legal, it was noted that most observers did not know this (Grant, 2016; Leeming et al., 2013; Morris et al., 2016; Pallotti, 2016; Ware et al., 2014), or they understood that breastfeeding in public was legal, but still perceived it to be inappropriate (Grant, 2016; Morris et al., 2016). Observers displaying support for the law were in a minority (Grant, 2015; Spurles & Babineau, 2011). One grandmother expressed in a mother/grandmother dyad interview that she thought breastfeeding in public was against the law (Pallotti, 2016).

### 3.6 Intersectional sexism, surveillance and stigma

The surveillance of women's bodies by strangers was regularly reported by pregnant women and mothers. All of the studies included in the analysis either explicitly reported on sexism and surveillance or contextualised their findings within an assumption of a sexist culture which used surveillance and stigma of women's bodies. Breasts were explicitly positioned as sexual, as opposed to maternal, particularly in studies reporting data from observers (Grant, 2015; Grant et al., 2017; Morris et al., 2016; Rhoden, 2016) and partners (Avery & Magnus, 2011; Bueno‐Gutierrez & Chantry, 2015; Chantry et al., 2008; Furman et al., 2013; Helps & Barclay, 2015; Henderson et al., 2011; Rhoden, 2016).

In addition to general sexist surveillance, some women were further marginalised by their intersectional characteristics (see Crenshaw, 1989) and reported this in relation to their views and experiences of breastfeeding in public when compared to white middle‐class women (Andrew & Harvey, 2011; Boyer, 2011, 2012, 2018; Leahy‐Warren et al., 2017; Leeming et al., 2013). In addition, midwives had additional knowledge arising from their profession which appeared to be a protective factor for some (Battersby, 2007). Racism and the perception of oneself being considered ‘out of place’ (as in Dowling and Pontin's [2017, p. 67] reference to breastmilk itself) was inferred in relation to Black women (Avery & Magnus, 2011; Furman et al., 2013; Owens et al., 2018; Robinson & VandeVusse, 2011; Ware et al., 2014) and their partners (Avery & Magnus, 2011; Rhoden, 2016); as well as Indigenous (Eni et al., 2014; Helps & Barclay, 2015), refugee and migrant (Chiang, 2017; Condon, 2018; Gallegos et al., 2015) women. Likewise, poverty and social class affected how pregnant women and mothers (Dyson et al., 2010; Grant et al., 2017; Shortt et al., 2013) as well as their partners (Henderson et al., 2011) considered breastfeeding in public, with the act seeming more acceptable in high‐income locations (Isherwood et al., 2019). Higher income also afforded individuals entry to breastfeeding‐friendly locations, such as cafes (Mathews, 2018). Young mothers also experienced feelings of heightened surveillance related to their age (Chopel et al., 2019; Nesbitt et al., 2012; Pallotti, 2016), as did women in larger bodies (Charlick et al., 2018; Hauck et al., 2020; McKenzie et al., 2018; Newman & Williamson, 2018). This was also experienced by more privileged women who breastfed older infants, particularly when infants were able to ask to breastfeed or to *help themselves* without asking (Andrew & Harvey, 2011; Dowling & Pontin, 2017; Hauck et al., 2020; Prendergast & James, 2016; Stearns, 2011; Swigart et al., 2017). Sexual orientation and gender identity were generally not reported.

#### 3.6.1 Societal discourses

Within this section, we consider the ways in which knowledge, beliefs, and the social environment (which is further divided into observers and the physical environment) impacted the experience, or perceived experience, of breastfeeding in public. Each of the discourses identified in the data was explored from the point of view of strangers/observers, those known to the mother and the mothers themselves.

### 3.7 Knowledge

In general, breastfeeding itself was viewed as good for infants’ health. However, there was evidence that demonstrated limited knowledge relating to normal infant feeding patterns and breastfeeding behaviours among observers (Grant, 2016; Rhoden, 2016), and partners (Avery & Magnus, 2011; Henderson et al., 2011). A lack of exposure to breastfeeding was associated with less knowledge among observers (Chiang, 2017; DeMaria et al., 2020; Jamie et al., 2020), whilst exposure increased comfort around breastfeeding in public for observers (DeMaria et al., 2020; Schmied et al., 2019), mothers (Chopel et al., 2019; Isherwood et al., 2019; Prendergast & James, 2016;), and fathers (Henderson et al., 2011). Examples of incorrect knowledge included observers believing that women decided when to feed a baby based on their preferences alone, and not that they responded to infants’ cues (Grant, 2015; Swigart et al., 2017) and the incorrect assumption that breastfeeding needed to expose the entire breast (Cato et al., 2020; Charlick et al., 2018). A small number of mothers reported limited knowledge of breastfeeding as a result of a lack of exposure to breastfeeding (Owens et al., 2018; Stearns, 2011). Additionally, breastmilk was viewed as a form of dangerous pollutant to be avoided at all costs by some participants, including observers, fathers, and mothers (Avery & Magnus, 2011; Grant, 2016; Morris et al., 2016; Spurles & Babineau, 2011).

In contrast to the limited knowledge of observers, many mothers used their experiential knowledge as a parent to highlight the *need* to respond to normal infant feeding cues to prevent infant distress (Battersby, 2007; Boyer, 2011; Brouwer et al., 2012; Charlick et al., 2017; Dyson et al., 2010; Eni et al., 2014; Grant, 2015; Leeming et al., 2013; Spurles & Babineau, 2011). In addition, in four studies (Boyer, 2012; Chiang, 2017; Gallegos et al., 2015; Grant et al., 2017) it was noted that mothers had previously lived in countries that were more supportive of breastfeeding in public spaces and that this influenced their exposure to, and knowledge of, breastfeeding before their own experience as a parent.

Attempts to increase knowledge occurred both through informal breastfeeding activism, known as *lactivism* (Prendergast & James, 2016) and two more formal interventions. First, the Australian Breastfeeding Welcome scheme, an intervention developed by the Australian Breastfeeding Association, provided stickers that venues staffed by employees with a ‘welcoming attitude’ and room to move a pram can use to declare ‘Breastfeeding Welcome Here’. Such venues can also be featured on an online list (Stevenson, 2019, p. 8). Second, a community‐based intervention in the United Kingdom displayed life‐size cardboard images of mothers breastfeeding, with the phrase ‘When breastfeeding is accepted, you won't look twice’, in venues including shopping centres and health centres. When evaluated, observers became more knowledgeable and exhibited increasingly favourable views towards breastfeeding (Condon et al., 2010; 29).

### 3.8 Societal beliefs

In every paper, we identified discourses suggesting that breastfeeding was viewed as an antisocial act to be conducted in private only; such discourses were largely related to the sexualisation of breasts. In two UK studies, breastfeeding was negatively associated with poverty and not being able to afford to purchase infant formula (Condon, 2018; Grant, 2016). However, in two studies reporting on social media content, these beliefs were contested (Lehto, 2019; Marcon et al., 2019). The most frequently reported emotional reaction arising from observing breastfeeding, within the context of it being considered an antisocial act, was discomfort (Battersby, 2007; Boyer, 2012; Grant, 2016; Henderson et al., 2011; Morris et al., 2016; Owens et al., 2018; Rhoden, 2016; Spurles & Babineau, 2011; Stearns, 2011). In two UK‐based studies (Grant, 2015; Morris et al., 2016), observers stated that they were concerned about being perceived by a breastfeeding woman as though they were ‘kind of perv’ (Grant, 2015, p. 145) or being viewed by other people as a ‘weirdo’ (Morris et al., 2016, p. 476). However, another observer suggested that ‘leering’ was a deliberate strategy to stop women breastfeeding outside of the home (Grant, 2015, p. 145). In three studies, female observers noted that *they* were not uncomfortable, but were concerned that men would be (Grant, 2016; Morris et al., 2016; Spurles & Babineau, 2011). Children were also shielded by women from images of breastfeeding in the United Kingdom (Henderson et al., 2011), and from viewing breastfeeding in Mexico (Bueno‐Gutierrez & Chantry, 2015).

Discomfort was rationalised in seven studies, due to breastmilk being a bodily fluid arising from a bodily function (Cato et al., 2020; Grant, 2015; Lehto, 2019; Mathews, 2018; Morris et al., 2016; Sheehan et al., 2019; Spurles & Babineau, 2011). To ease their feelings of discomfort, observers suggested that discretion should be used by breastfeeding mothers in five studies (Grant, 2016; Morris et al., 2016; Rhoden, 2016; Spurles & Babineau, 2011; Ware et al., 2014), such as using a ‘cover’ whilst breastfeeding (Rhoden, 2016, p. 161); a ‘designated room’ (Spurles & Babineau, 2011, p. 134) or a ‘private’ space (Grant, 2016, p. 56).

Disapproval from partners was identified in 11 studies (Avery & Magnus, 2011; Brouwer et al., 2012; Carlin et al., 2019; Chantry et al., 2008; Dayton et al., 2019; Eni et al., 2014; Furman et al., 2013; Helps & Barclay, 2015; Henderson et al., 2011; Sheehan et al., 2019; Ware et al., 2014), with a complex range of concepts informing the disapproval. Occasionally, a difficulty reconciling breasts with infant feeding resulted in disgust reactions (Furman et al., 2013; Helps & Barclay, 2015) where breastfeeding was referred to as ‘nasty…freaky stuff’ (Furman et al., 2013, p. 62). More usually, breastfeeding itself was not the problem, but the social context whereby other men may view the individual's partner sexually led to two reactions. First, partners expressed concern over the potential for them to be involved in a confrontation with strangers (Avery & Magnus, 2011; Henderson et al., 2011). Second, male partners reported feelings of ownership over their partners’ bodies (Chantry et al., 2008; Furman et al., 2013; Helps & Barclay, 2015; Henderson et al., 2011). Explicit concern regarded their partners’ use of public transport coinciding with a need to breastfeed was described by young fathers (Henderson et al., 2011). In addition, other family members were identified as a source of disapproval in six studies (Boyer, 2012; Eni et al., 2014; Newman & Williamson, 2018; Pallotti, 2016; Rhoden, 2016; Sheehan et al., 2019), with friends (DeMaria et al., 2020) and health professionals (Llorente‐Pulido et al., 2021) also identified as disapproving.

### 3.9 Social environment: People

Negative beliefs associated with breastfeeding in public translated into individual mothers being discouraged from breastfeeding in public by partners (Dayton et al., 2019; Isherwood et al., 2019; Sheehan et al., 2019; Stav, 2019) and family (Isherwood et al., 2019; Newman & Williamson, 2018; Prendergast & James, 2016; Sheehan et al., 2019; Stav, 2019). Contrastingly, some partners (Alianmoghaddam et al., 2017; Hauck et al., 2020; Leeming et al., 2013; Majee et al., 2017; Owens et al., 2018; Pallotti, 2016; Shortt et al., 2013; Swigart et al., 2017), family members (Alianmoghaddam et al., 2017; Hauck et al., 2020; Majee et al., 2017; Schafer et al., 2019; Sheehan et al., 2019), and friends (Alianmoghaddam et al., 2017; Chopel et al., 2019; DeMaria et al., 2020; Hauck et al., 2020; Schafer et al., 2019) were supportive of breastfeeding in public. Staff working in public places both encouraged (Hauck et al., 2020; Mathews, 2018; Schmied et al., 2019) and discouraged (Chopel et al., 2019; Mathews, 2018; McKenzie et al., 2018) breastfeeding in public.

The most considered social aspect within the included papers was in relation to strangers. It was commonly reported, usually by mothers, that some observers behaved in hostile ways towards breastfeeding mothers. This included negative looks (Boyer, 2012; Chantry et al., 2008; Chopel et al., 2019; Eni et al., 2014; Forster & McLachlan, 2010; Hauck et al., 2020; Isherwood et al., 2019; Llorente‐Pulido et al., 2021; Majee et al., 2017; Marsden & Abayomi, 2012; Mathews, 2018; McKenzie et al., 2018; Owens et al., 2018; Pallotti, 2016; Sheehan et al., 2019; Thomson et al., 2015; Ware et al., 2014; Zhou et al., 2020); gestures (Boyer, 2012; Mathews, 2018; Shortt et al., 2013; Stearns, 2011; Thomson et al., 2015); ‘tuts of disgust’ (Shortt et al., 2013, p. 456; Thomson et al., 2015); and making negative comments (Chopel et al., 2019; DeMaria et al., 2020; Jamie et al., 2020; Lehto, 2019; Shortt et al., 2013), including to young mothers (Nesbitt et al., 2012). The intention behind looks, gestures, tuts and comments was not always clear, but they contributed to mothers' sense of discomfort about breastfeeding in public spaces. Three interactions were also reported where mothers who were breastfeeding had their personal space invaded by strangers, including one man behaving sexually towards a woman on a bus (Furman et al., 2013), a stranger masturbating near a breastfeeding woman (Lehto, 2019), and an older woman who ‘ripped off’ a young mothers' breastfeeding cover on a sunny day (Pallotti, 2016, p. 158). An African American woman also noted sexualised reactions when she was breastfeeding in public (Owens et al., 2018).

Occasionally it was reported that mothers were (Thomson et al., 2015), or would be (Pallotti, 2016), asked to leave the premises because they were breastfeeding, typically as a result of observers complaining to members of staff (Battersby, 2007). Staff in public spaces, such as restaurants and shops, were discussed in four papers by mothers and observers, all from the United Kingdom (Battersby, 2007; Grant, 2015; Grant et al., 2017; Morris et al., 2016). Reports included those where staff had been awkward (Battersby, 2007) or asked intrusive questions (Grant et al., 2017), and there were media reports of instances where staff had denied women their legal right to breastfeed (Grant, 2015; Morris et al., 2016). One study (Marsden & Abayomi, 2012) reported on interviews with staff working in public spaces. In these interviews, staff recounted instances of observers displaying disapproval of breastfeeding. The authors concluded that the experience of being a parent or working in a ‘baby friendly’^1^ space led to staff members displaying increased confidence in supporting breastfeeding mothers.

Reports of positive interactions with strangers whilst breastfeeding in public were rare, but included polite inattention (Majee et al., 2017), encouraging gestures (Hauck et al., 2020; O'sullivan et al., 2020; Prendergast & James, 2016) and kind comments (Alianmoghaddam et al., 2017; Dowling & Pontin, 2017; Jamie et al., 2020; Mathews, 2018; O'sullivan et al., 2020). Additionally, one article which focused on middle‐class mothers in the United Kingdom reported that: ‘not all women encountered social opprobrium for breastfeeding in public’, highlighting that one mother reported receiving more positive comments from strangers about her baby ‘than any kind of negative feeling about breastfeeding’ (Boyer, 2012, p. 559).

### 3.10 Social environment: Physical environment

Alongside a challenging social context, the physical environment was reported to be generally lacking in comfortable spaces to breastfeed (Battersby, 2007; Boyer, 2012; Brouwer et al., 2012; Charlick et al., 2017; Eni et al., 2014; Forster & McLachlan, 2010; Grant, 2021; Hauck et al., 2020; Isherwood et al., 2019; Mathews, 2018; O'sullivan et al., 2020; Owens et al., 2018; Schmied et al., 2019; Shortt et al., 2013; Stav, 2019; West et al., 2017). Criticisms included a lack of places to sit (Battersby, 2007; Forster & McLachlan, 2010; Grant, 2021; Hauck et al., 2020; Isherwood et al., 2019; O'sullivan et al., 2020), overly bright lighting (Boyer, 2012), and a lack of privacy (Brouwer et al., 2012; Leahy‐Warren et al., 2017; Shortt et al., 2013). This led to individuals not feeling emotionally comfortable (Chiang, 2017; Isherwood et al., 2019; Sheehan et al., 2019; Swigart et al., 2017; Zhou et al., 2020).

A range of places were identified as inappropriate for breastfeeding, including: shops (Charlick et al., 2017; Chopel et al., 2019; Furman et al., 2013; Grant, 2016; Newman & Williamson, 2018; Owens et al., 2018; Pallotti, 2016; Sheehan et al., 2019; Spurles & Babineau, 2011); public transport (Battersby, 2007; Dyson et al., 2010; Furman et al., 2013; Henderson et al., 2011; Marsden & Abayomi, 2012; Sheehan et al., 2019) and places where people eat (Charlick et al., 2017; DeMaria et al., 2020; Grant et al., 2019; Leeming et al., 2013; Marsden & Abayomi, 2012; Newman & Williamson, 2018; Owens et al., 2018; Sheehan et al., 2019; Shortt et al., 2013; Spurles & Babineau, 2011). Less frequently described areas of concern included events held at schools (Chopel et al., 2019; Newman & Williamson, 2018; Stav, 2019), church (Chopel et al., 2019; Newman & Williamson, 2018) and parliament (Sheehan et al., 2019).

Physical spaces that facilitated breastfeeding were identified as having comfortable seating (Boyer, 2012; Hauck et al., 2020; Stav, 2019), as well as a degree of privacy (Hauck et al., 2020; Sheehan et al., 2019; Stav, 2019), cosiness and the presence of other families with young children (Stav, 2019). This could include cafes and restaurants which warmly welcomed breastfeeding (Chopel et al., 2019; Newman & Williamson, 2018; Schmied et al., 2019; Stevenson, 2019), including the use of ‘Breastfeeding Welcome’ stickers (Hauck et al., 2020; Isherwood et al., 2019; Schmied et al., 2019; Stevenson, 2019). However, one author noted that not all mothers were financially able to use these spaces (Mathews, 2018). The ‘Feed Finder’ app was used to identify safe places in some UK‐based studies (Grant, 2021; Isherwood et al., 2019). Furthermore, a supportive online environment sometimes helped women to gain confidence in offline situations (Hauck et al., 2020; Lehto, 2019; Robinson & VandeVusse, 2011).

### 3.11 Social environment: Mother and baby rooms V toilets

Mother and baby rooms, generally located within shopping centres (with one room noted on a college campus in Rose, 2012) were identified both positively and negatively. They reportedly provided a ‘private’ space away from home (Battersby, 2007; Boyer, 2012; Brouwer et al., 2012; Charlick et al., 2017; Charlick et al., 2018; O'sullivan et al., 2020; Zhou et al., 2020), which was especially valued in the early weeks of breastfeeding (Boyer, 2012; Brouwer et al., 2012). Mother and baby rooms identified as having high‐quality facilities were particularly viewed positively (Hauck et al., 2020), although even low‐quality facilities were sometimes viewed as better than no provision at all (Schmied et al., 2019).

Conflictingly, mother and baby rooms were viewed by some as isolating (Battersby, 2007; Boyer, 2012), and were sometimes inaccessible due to lack of availability in some locations (McKenzie et al., 2018), existing facilities being busy (Grant, 2021; Schmied et al., 2019), and facilities being locked (Grant, 2021; Mathews, 2018) or hidden (Grant, 2021; Schmied et al., 2019). Mother and baby facilities could also be unpleasant (Isherwood et al., 2019) because they were, for example, often positioned in or near toilets or baby changing areas (Battersby, 2007; Boyer, 2012) which caused disgusting smells (Grant, 2021; Schmied et al., 2019). Poor design was also reported (Boyer, 2012), including inadequate furniture (Grant, 2021) and lighting (Mathews, 2018). Designated mother and baby spaces sometimes felt unsafe due to use by other groups, including substance users (Mathews, 2018; Schmied et al., 2019).

The presence of spaces designated for breastfeeding could make other spaces appear unsuitable for breastfeeding to members of the public (Brouwer et al., 2012; Marsden & Abayomi, 2012; Spurles & Babineau, 2011). This sentiment was echoed by one midwife who was also a mother (Battersby, 2007). Observers relatively frequently identified toilet cubicles as a place to breastfeed or express breast milk (Avery & Magnus, 2011; Brouwer et al., 2012; Grant, 2016; Helps & Barclay, 2015; Leahy‐Warren et al., 2017; Leeming et al., 2013; Marsden & Abayomi, 2012; Robinson & VandeVusse, 2011; Shortt et al., 2013; Spurles & Babineau, 2011; West et al., 2017). However, mothers (Brouwer et al., 2012; Grant, 2015; Leeming et al., 2013; Robinson & VandeVusse, 2011; Shortt et al., 2013; West et al., 2017) and partners (Marsden & Abayomi, 2012) reported that public toilets were dirty and the experience unpleasant. There were no positive experiences reported of breastfeeding in a toilet.

#### 3.11.1 Mothers' response to societal barriers and facilitators

In this section, we divide mothers' responses to the wider social context and societal discourses into their thoughts and behaviour, which arose in response to the social environment within which they existed.

### 3.12 Mothers’ thoughts

In all but 7 of the 63 studies that included the views of mothers and/or pregnant women, the societal belief that breastfeeding should be a ‘private’ activity was firmly embedded. This belief was more prominent in mothers of older infants (Isherwood et al., 2019; Mathews, 2018; Newman & Williamson, 2018; Prendergast & James, 2016; Swigart et al., 2017). This was usually tied to an understanding of hostile societal beliefs and an associated unpleasant environment in terms of the physical space and the potential for conflict from strangers. Some mothers explained their negative thought processes relating to breastfeeding in public as directly originating from family (Boyer, 2011; Eni et al., 2014; Furman et al., 2013; Helps & Barclay, 2015), friends (Boyer, 2011; Eni et al., 2014; Owens et al., 2018) and strangers (Boyer, 2012; Gallegos et al., 2015; Helps & Barclay, 2015). Mothers and pregnant women reported anticipating being disapproved of in 27 papers. Within this context, it is unsurprising that women in many papers felt embarrassed, uncomfortable, self‐conscious, and exposed, as is illustrated in Table 4.

**Table 4:** Mothers' negative feelings relating to their own experience of breastfeeding in public

Theme	Subtheme	Studies
**Positive**	No issues	(Charlick et al., 2019; Chopel et al., 2019; Isherwood et al., 2019; Schafer et al., 2019; Sheehan et al., 2019; Stevenson, 2019).
**Changing**	‘Got used to it’	(Battersby, 2007; Boyer, 2012; Charlick et al., 2018; Charlick et al., 2019; Forster & McLachlan, 2010; Isherwood et al., 2019; McKenzie et al., 2018; Prendergast & James, 2016).
**Negative**	Uncomfortable	(Andrew & Harvey, 2011; Battersby, 2007; Boyer, 2012; Brouwer et al., 2012; Bueno‐Gutierrez & Chantry, 2015; Charlick et al., 2018, 2019; Chiang, 2017; Dowling & Pontin, 2017; Dyson et al., 2010; Eni et al., 2014; Forster & McLachlan, 2010; Grant et al., 2017; Helps & Barclay, 2015; Isherwood et al., 2019; Lee, 2019; Jamie et al., 2020; Marsden & Abayomi, 2012; Mathews, 2018; McKenzie et al., 2018; Nesbitt et al., 2012; Owens et al., 2018; Pallotti, 2016; Robinson et al., 2019; Sheehan et al., 2019; Stav, 2019; Thomson et al., 2015; West et al., 2017).
	Self‐conscious	(Battersby, 2007; Boyer, 2012; Brouwer et al., 2012; Chiang, 2017; Charlick et al., 2017; Charlick et al., 2018; Dyson et al., 2010; Forster & McLachlan, 2010; Grant et al., 2017; Grant et al., 2019; Hauck et al., 2021; Hinson et al., 2018; Jamie et al., 2020; Leahy‐Warren et al., 2017; Leeming et al., 2013; Mathews 2018; McKenzie et al., 2018; Nesbitt et al., 2012; Newman & Williamson, 2018; O'Sullivan et al., 2020; Owens et al., 2018; Pallotti, 2016; Prendergast & James, 2016; Robinson & VandeVusse, 2011; Shortt et al., 2013; Stav, 2019; Thomson et al., 2015; Ware et al., 2014; West et al., 2017; Zhou et al., 2020).
	Embarrassed	(Battersby, 2007; Boyer, 2012; Brouwer et al., 2012; DeMaria et al., 2020; Dyson et al., 2010; Eni et al., 2014; Forster & McLachlan, 2010; Gallegos et al., 2015; Grant et al., 2017; Hauck et al., 2020; Helps & Barclay, 2015; Hinson, et al., 2018; Leahy‐Warren et al., 2017; Nesbitt et al., 2012; Owens et al., 2018; Pallotti, 2016; Prendergast & James, 2016; Robinson & VandeVusse, 2011; Sheehan, et al., 2019; Shortt et al., 2013; Stav, 2019; Swigart et al., 2017).
	Exposing/sexual	(Brouwer et al., 2012; Bueno‐Gutierrez & Chantry, 2015; Cato et al., 2020; Charlick et al., 2018; Chopel, et al., 2019; Dyson et al., 2010; Eni et al., 2014; Forster & McLachlan, 2010; Grant et al., 2017; Hauck et al., 2021; Jamie et al., 2020; Leeming et al., 2013; Mathews, 2018; McKenzie et al., 2018; Pallotti, 2016; Robinson & VandeVusse, 2011; Sheehan et al., 2019; Stav, 2019).
	Ashamed	(Chopel, et al. 2019; Forster & McLachlan, 2010; Gallegos et al., 2015; Hauck, et al., 2020; Helps & Barclay, 2015; Lehto, 2019; Owens et al., 2018).
	Worried/anxious	(Boyer, 2012; Brouwer et al., 2012; Charlick, et al., 2018; Charlick, et al., 2019; Grant et al., 2017; Hauck et al., 2020; Isherwood et al., 2019; Jamie et al., 2020; Lee, 2019; Mathews, 2018; McKenzie et al., 2018; Nesbitt et al., 2012; Newman & Williamson, 2018; Sheehan et al., 2019; Stav, 2019; Stevenson, 2019; Swigart et al., 2017).
	Traumatic	(Battersby, 2007; Boyer, 2012; Forster & McLachlan, 2010; Grant et al., 2017).
	Fearful	(Boyer, 2012; Forster & McLachlan, 2010; Hauck, et al., 2020; Isherwood et al., 2019; McKenzie et al., 2018; Thomson et al., 2015; Stav, 2019).
	Paranoid	(Forster & McLachlan, 2010; McKenzie et al., 2018).

Mothers noted the need to be ‘discreet’ as a form of protection against visibly negative responses and confrontation that could arise from breastfeeding in public, including perceived physical and sexual threats (Battersby, 2007; Boyer, 2011; Chantry et al., 2008; Charlick et al., 2017; Grant et al., 2017; Helps & Barclay, 2015; Leahy‐Warren et al., 2017; Leeming et al., 2013; Lehto, 2019; Newman & Williamson, 2018; Owens et al., 2018; Sheehan et al., 2019; Stearns, 2011; Thomson et al., 2015). In addition to considering the *need* for discretion in their own behaviour, some mothers who breastfeed in public identified other women as breastfeeding in public in a less appropriate way than they did (Boyer, 2011; Bueno‐Gutierrez & Chantry, 2015; Chantry et al., 2008; Charlick et al., 2017; Charlick et al., 2018; Chopel et al., 2019; Grant, 2016; Jamie et al., 2020; Leeming et al., 2013; Pallotti, 2016; Sheehan et al., 2019). This included exposing more skin than was deemed essential (Chantry et al., 2008; Charlick et al., 2017; Grant, 2016; Grant et al., 2017) or being seen to be making a political point (Boyer, 2011; Leeming et al., 2013). In rare cases, breastfeeding in public was viewed by other mothers as inappropriate; or ‘sick and twisted’ (Dyson et al., 2010, p. 146).

Conversely, a small minority of mothers reported feeling empowered by breastfeeding in public (Battersby, 2007; Boyer, 2012; Robinson et al., 2019). We inferred that a culture of intensive motherhood, where infant demands were prioritised above maternal comfort, provided an opposing counter‐pressure and rationale for breastfeeding regardless of a hostile social context (Charlick et al., 2017; Condon et al., 2010; Dyson et al., 2010; Furman et al., 2013; Gallegos et al., 2015; Grant, 2015, 2016; Grant et al., 2017; Marsden & Abayomi, 2012; Stearns, 2011; Ware et al., 2014), particularly in mothers who were not strongly marginalised as a result of their demographics (Battersby, 2007; Dowling & Pontin, 2017). Some mothers and, to a lesser extent family, subscribed to a view that babies' needs should be met on demand (Battersby, 2007; Boyer, 2011; Brouwer et al., 2012; Charlick et al., 2017, 2018; Condon et al., 2010; Dyson et al., 2010; Eni et al., 2014; Grant, 2016; Leeming et al., 2013; Spurles & Babineau, 2011). Furthermore, the potential for babies to cry and disturb observers was noted as a particular justification for breastfeeding in public spaces by some mothers (Andrew & Harvey, 2011; Battersby, 2007; Cato et al., 2020; Charlick et al., 2019; Grant, 2016; Hauck et al., 2020; Marsden & Abayomi, 2012; Mathews, 2018; Swigart et al., 2017).

### 3.13 Mothers' behaviour

In 37 studies that reported women's experiences, at least some of the participants did not breastfeed in public. Conversely, participants in six papers reported that they were able to breastfeed in public without issue, with mothers in eight papers noting that they ‘got used to’ breastfeeding in public as they became more experienced. Participants who reported neutral or positive accounts included those who were multi‐parous (Andrew & Harvey, 2011; Battersby, 2007), more experienced at breastfeeding (Battersby, 2007), had supportive partners (Alianmoghaddam et al., 2017), were from relatively privileged, white middle‐class, backgrounds in the United Kingdom (Boyer, 2012; Dowling & Pontin, 2017), had been exposed to a breastfeeding in public intervention (Stevenson, 2019), or had been part of online breastfeeding groups which engendered greater confidence (Robinson et al., 2019). Five participants responding to a survey noted ‘no negative view’ (Forster & McLachlan, 2010, p. 121), with a quarter of respondents in another study noting that breastfeeding *facilitated* socialising outside of the home, due to its greater convenience than bottle feeding (Nesbitt et al., 2012). Additionally, ‘community breastfeeding champions’ were identified as ‘(achieving) breastfeeding success through their…lack of concern for the opinions of others…’ (Helps & Barclay, 2015, p. 133).

In the majority of studies reporting mothers' experiences of breastfeeding outside of the home (*n* = 49), women noted that they breastfed in a highly self‐aware way to protect themselves from the hostile social environment, and also to protect observers from potential discomfort; we termed this ‘doing it anyway’. This involved actualising the need to be ‘discrete (sic)’ through a range of strategies (Leahy‐Warren et al., 2017, p. 106) including the use of clothing (Carlin et al. 2019; Charlick et al., 2018; Grant et al., 2017; Hauck et al., 2020; McKenzie et al., 2018; Newman & Williamson, 2018), shawls (Battersby, 2007; Charlick et al., 2017; Furman et al., 2013; Gallegos et al., 2015; Grant et al., 2017; Robinson & VandeVusse, 2011; West et al., 2017) or specific breastfeeding covers (Charlick et al., 2018; DeMaria et al., 2020; Eni et al., 2014; Hauck et al., 2020; McKenzie et al., 2018; Owens et al., 2018; Pallotti, 2016; Schindler‐Ruwisch et al., 2019; Schmied et al., 2019; Sheehan et al., 2019; Swigart et al., 2017) to hide the maternal breast. Two mothers noted panicking when their chosen shawl/cover had been left at home and their baby needed feeding in public; both noted the support of their partners during this single feed (Grant et al., 2017; Owens et al., 2018). Only one mother reported that she refused to use a cover, due to it being hot and reducing the visibility of her baby (Owens et al., 2018).

Having large breasts (Battersby, 2007; Grant et al., 2017), an excess milk supply leading to leakage (Leeming et al., 2013), or being inexperienced in the mechanics of breastfeeding (Boyer, 2011; Brouwer et al., 2012), were all identified as additional challenges in hiding breastfeeding. Infants could also contribute to making breastfeeding more visible, including a baby who was ‘off and on’ the breast (Leeming et al., 2013, p. 463; Owens et al., 2018) or noisy during feeding. One mother noted avoiding making eye contact with strangers to prevent inadvertently opening an opportunity for interaction (Dowling & Pontin, 2017), whilst others moved themselves into a more private area (Charlick et al., 2017).

Despite many women's self‐conscious behaviour and attempts for discretion, experiencing negative feedback from observers resulted in some women never attempting to breastfeed in public again (Helps & Barclay, 2015; Pallotti, 2016). As a consequence, some women therefore reported providing their infants with infant formula (Andrew & Harvey, 2011; Eni et al., 2014; Forster & McLachlan, 2010; Grant et al., 2017; Leeming et al., 2013; Owens et al., 2018; Robinson & VandeVusse, 2011) or expressed breastmilk (Grant et al., 2017; Leeming et al., 2013), or undisclosed ‘milk’ from a bottle (Battersby, 2007; Boyer, 2012; Dyson et al., 2010; Helps & Barclay, 2015; Nesbitt et al., 2012; Pallotti, 2016) in public spaces. In one case, a woman noted that negative feedback from breastfeeding in public was the reason for her stopping breastfeeding earlier than she wanted to, when her baby was 3 weeks old (Boyer, 2012). By contrast, lockdowns associated with the COVID‐19 pandemic in the United Kingdom were associated with providing more privacy which enabled women to avoid public breastfeeding, but also resulted in some mothers lacking the skills and confidence to breastfeed in public once lockdowns were removed (Brown & Shenker, 2021).

Other mothers who continued breastfeeding moved to only using a ‘private’ or semi‐private space (Nesbitt et al., 2012; Owens et al., 2018; Shortt et al., 2013; Thomson et al., 2015) where available, including mother and baby rooms (Battersby, 2007; Boyer, 2012; Brouwer et al., 2012; Charlick et al., 2017; Helps & Barclay, 2015; Spurles & Babineau, 2011) and toilets (Helps & Barclay, 2015; Leahy‐Warren et al., 2017; Leeming et al., 2013; Marsden & Abayomi, 2012; Robinson & VandeVusse, 2011; Shortt et al., 2013; West et al., 2017). Some mothers reported using their cars as a place to breastfeed when away from home, and when it was parked in a sufficiently discreet place (Furman et al., 2013; Helps & Barclay, 2015; Leahy‐Warren et al., 2017; Leeming et al., 2013; Robinson & VandeVusse, 2011; Shortt et al., 2013; Spurles & Babineau, 2011; West et al., 2017). This was not reported to be a satisfactory solution in any of the papers, and it was explicitly stated that this was unpleasant or inconvenient in several instances (Furman et al., 2013; Shortt et al., 2013; West et al., 2017). A further protective strategy adopted by a minority of mothers was to only breastfeed in public in the company of other breastfeeding mothers (Charlick et al., 2018; Isherwood et al., 2019; Newman & Williamson, 2018; Stav, 2019) or on days when they felt able to cope with a potential confrontation (Mathews, 2018). In very rare instances, women confronted those who responded negatively to their breastfeeding, including one woman whose neighbour criticised her breastfeeding in front of her (Leahy‐Warren et al., 2017).

The alternative behaviour described by mothers and pregnant women to hide the maternal breast in public spaces was for breastfeeding mothers to stay at home (Boyer, 2011; Forster & McLachlan, 2010; Leeming et al., 2013; Owens et al., 2018), which was isolating (Andrew & Harvey, 2011; Dyson et al., 2010; Nesbitt et al., 2012; Pallotti, 2016; Thomson et al., 2015), bad for maternal mental health (Andrew & Harvey, 2011; Boyer, 2018; Nesbitt et al., 2012) and difficult if mothers had older children (Andrew & Harvey, 2011). Women breastfeeding older infants sometimes restricted breastfeeding to the home (Dowling & Pontin, 2017; Stearns, 2011).

## 4 DISCUSSION

Our thematic synthesis of 71 studies, reported in a total of 74 papers, covered over 17,000 mothers from 12 OECD countries. We identified five areas that influenced mothers' thoughts and behaviour in relation to breastfeeding in public: legal systems, intersectional inequality, knowledge, beliefs, and the social environment. Each of these themes contained barriers and facilitators, although limited attention was paid to the macro‐level influences that we identified—the legal system and structural inequality. Furthermore, the attention of observers was firmly focused on mothers as sexualised women, rather than as caregivers to infants who needed food within the mother‐baby dyad. We strongly identified anti‐breastfeeding beliefs in the majority of the members of the public within the qualitative studies included in this review, which we inferred as originating from lack of knowledge of the needs of infants in relation to feeding and normal breastfeeding behaviour, alongside the sexualisation of breasts and the mistaken belief that breastmilk was a biohazard. Partners, family and friends were sometimes supportive, but other times discouraged breastfeeding in public. Limited research had been undertaken on the views of staff working in public spaces but, within the included studies, staff did not feel fully comfortable supporting breastfeeding in their workplace.

Mothers identified that the built environment often resulted in no ‘good’ place to breastfeed, although places with comfortable seating and a ‘safe’ feeling atmosphere were identified in a minority of studies. Additionally, mothers were mixed in their opinions regarding the value of mother and baby rooms, which could feel supportive, but were also inaccessible and unsuitable in many ways. The majority of mothers felt negatively about breastfeeding in public and anticipated conflict. Many reported negative behaviours directed towards them or other mothers they knew when breastfeeding in public including looks, tuts, negative comments and occasional touching. Positive experiences were described much less frequently. As a response, mothers avoided breastfeeding in public, or did so ‘discreetly’ using clothing and covers designed to minimise the view to strangers. Maternal knowledge of legal rights protecting breastfeeding appeared to be a facilitator for breastfeeding in public in a small number of studies.

Our synthesis highlighted a large range of barriers, and rather fewer facilitators, to breastfeeding in public. Within this context, mothers' avoidant and highly self‐aware breastfeeding in public behaviour should be viewed as a functional and protective response to a hostile environment, in urgent need of change. Existing interventions included: peer supporters spreading knowledge (Condon et al., 2010), The Breastfeeding Welcome scheme (Stevenson,  2019), and approaches using crowd‐sourced information and mapping technology to find ‘supportive’ (Simpson et al., 2016, p. 2) or hidden spaces for breastfeeding (Shankar et al., 2019). Based on our thematic synthesis, however, we believe that changes to the built environment alone, such as the use of pods and mother and baby rooms, may undermine breastfeeding in public in a wider sense by hiding it from public view (Battersby, 2007), and thus we do not recommend this strategy. Accordingly, instead of directing interventions towards mothers who exist in a hostile context, the narrative around breastfeeding in public should be reframed around the needs of the baby, as facilitated by its mother, rather than as something the mother does to meet her own needs. Theoretically informed interventions should be developed to spread this narrative among the public (Skivington et al., 2021).

Core elements that should be addressed by interventions include increasing knowledge among the general public to reduce the continuing presence of negative beliefs suggesting that breastfeeding is sexual, and that breastmilk is a contamination threat. This would directly aim to reframe beliefs centred around mothers' sexuality, illuminating the importance of breastfeeding in public for babies’ nutrition. Social media campaigns may be of value (Giles, 2018), as well as changes in legislation and enforcing existing rights to breastfeed contained in legislation. We hypothesise that when the social environment is more welcoming, the limitations of the built environment will have less impact on where mothers feel that they can breastfeed. It is well established that appropriately tailored face‐to‐face support provided to breastfeeding mothers increases breastfeeding duration and exclusivity (McFadden et al., 2017). These principles could be used in relation to building confidence to breastfeed in public, alongside more general support on how to physically breastfeed.

Feminist theories, used in several included studies, explain the social discomfort around breastfeeding in public as arising from a patriarchal society which is not appropriately set up to meet the needs of women, let alone mothers (Ahmed, 2017). This particularly affects mothers from marginalised groups, including young, Black, and Disabled women, who can be further stigmatised (Crenshaw, 1989). This discomfort has been explained using a wide range of mid‐range theories, including Goffman's social interaction (Brouwer et al., 2012), liminality (Dowling & Pontin, 2017), Sara Ahmed's affect (Boyer, 2012), Foucault's heterotopian space (Rose, 2012), Lazare's shame (Thomson et al., 2015), power and poverty (Groleau et al., 2013), and Heidegger's concept of they (McBride‐Henry, 2010). In addition, mothers themselves, in attempting to maintain their own ‘good mother’ identity, were involved in generating and sustaining stigma in relation to those who breastfed in a way that was perceived as being less modestly than they did. The phenomenon of ‘othering’, identified and linked to the generation of stigma more than half a century ago (Goffman, 1959), has already been considered in one paper (Brouwer et al., 2012), and is worthy of further exploration within the context of breastfeeding in public. Collectively, this theorising highlights the importance of power and stigma in guiding infant feeding in public views and behaviours, and feminist theories may be valuable in developing new interventions.

### 4.1 Strengths and weaknesses

Our analysis of 71 studies from 2007 to May 2021 followed rigorous systematic review and evidence synthesis (Thomas & Harden, 2008) procedures through a series of meetings between a team of infant feeding researchers from varying disciplinary backgrounds. The synthesis highlighted considerably more barriers to breastfeeding in public than facilitators. This may be because the majority of papers included were concentrated in three high‐income counties—United Kingdom, United States and Australia—within the 38 OECD countries. Countries where breastfeeding rates are considerably higher than, for example, the United Kingdom (such as the Nordic countries) were not included in our systematic search. This could be because of the English language focus of our systematic literature search, or the lack of problematisation of breastfeeding in public in countries with higher breastfeeding rates. In addition, our restriction to only journal articles and books will likely have excluded grey literature which could have shed additional light on interventions available at national and local levels within OECD countries. Whilst research was included that focused on marginalised groups including Black, Indigenous, Fat, young and low‐income women, there was very limited content identified as originating from mothers of minority sexual orientations, and no content from trans men, nonbinary, and other minority gender identities for whom breastfeeding (or chestfeeding) in public may be further stigmatised. Finally, only a small amount of evidence was included based on perceptions of ‘safe’ spaces to breastfeed, and there is an urgent need for further research into women's views and experiences of positive breastfeeding in public experiences.

## 5 CONCLUSION

Our systematic review of experiences of women from OECD countries has identified a wide range of barriers to breastfeeding in public spaces. Mothers’ thoughts and behaviour in relation to breastfeeding in public are often a functional and protective response to embarrassment, discomfort, shame, and anxiety resulting from a hostile social environment. Breastfeeding is a gendered behaviour and exists within a patriarchal culture where gendered stigma interacts with other characteristics such as racism, ageism, and classism. Less marginalised mothers are known to breastfeed for longer which may be due to them experiencing society as less stigmatising in relation to breastfeeding. Interventions to promote breastfeeding in public—particularly focused on improving legal support, increasing societal knowledge, and decreasing stigma associated with the maternal breast—may hold promise. As with much of the research related to breastfeeding, there is an urgent need for appropriately funded investment to develop and evaluate interventions that specifically target the social and physical environment, rather than focusing solely on individual‐level interventions that target mothers' (rational) beliefs, knowledge and attitudes.

## AUTHOR CONTRIBUTIONS

Aimee Grant developed the application for funding and the scope of the review (with guidance from Michael Robling). Aimee Grant led the review, taking part in all aspects of it. Delyth Morris provided support with the development of the search strategy and undertook the searches. Double screening of titles and abstracts was undertaken by Aimee Grant, Rebecca Ellis (and Michael Robling). Double screening of full texts was undertaken by Aimee Grant, Bethan Pell and Rebecca Ellis. Double screening of texts for quality (using the CASP tool) was undertaken by Aimee Grant and Lauren Copeland. Double coding of a selection of papers was undertaken by Rhiannon Phillips. Bethan Pell, Lauren Copeland, Amy Brown, Denitza Williams and Rhiannon Phillips attended a series of data analysis workshops chaired by Aimee Grant. Aimee Grant wrote the paper. All authors reviewed the manuscript.

## CONFLICTS OF INTEREST

AG: AG's position when undertaking this Fellowship was funded by the Wellcome Trust. Her current position is funded by the Higher Education Council for Wales (HEFCW). BP: BP's position at the time of the project was funded by the National Institute for Health Research. Her current position is funded by Health and Care Research Wales. She has recently been awarded an ESRC DTP Studentship which will start in October 2021. LC: LC's position at the time of the project was funded by Health and Care Research Wales. Her current position is funded by National Institute for health Research; and Health and Care Research Wales. AB: Is Director of the Centre for Lactation, Infant Feeding and Translational Research. Her time on this project was funded by the Higher Education Council for Wales (HEFCW). RE: Is a Health and Care Research Wales funded PhD student. Her time on this project was funded by the Higher Education Council for Wales (HEFCW) through the Centre for Lactation, Infant Feeding and Translational Research. DM & DW: No conflicts of interest. RP: At the time the first phase of this project was undertaken, RP's PRIME Centre Wales fellowship was funded by Health and Care Research Wales.

## Data Availability

This study was a systematic review that did not produce any new data. Accordingly, there is no data to be made available.

## Footnotes

1 The UNICEF Baby Friendly Initiative (BFI) is a programme of guidance and accreditation for maternity services. It is not clear if the ‘baby friendly’ businesses were chosen because of their affinity to the BFI initiative.

## APPENDIX A MEDLINE SEARCH STRATEGY

**Table:**

1. exp Breast Feeding/
2. (infant feed* or breast feed* or breastfeed* or breast‐feed* or breast fed or infant fed).mp. [mp=title, abstract, original title, name of substance word, subject heading word, keyword heading word, protocol supplementary concept word, rare disease supplementary concept word, unique identifier, synonyms]
3. Milk, Human/
4. 1 or 2 or 3
5. exp Social Environment/
6. exp Social Norms/
7. public space*.mp. [mp=title, abstract, original title, name of substance word, subject heading word, keyword heading word, protocol supplementary concept word, rare disease supplementary concept word, unique identifier, synonyms]
8. public environment*.mp. [mp=title, abstract, original title, name of substance word, subject heading word, keyword heading word, protocol supplementary concept word, rare disease supplementary concept word, unique identifier, synonyms]
9. social environment*.mp. [mp=title, abstract, original title, name of substance word, subject heading word, keyword heading word, protocol supplementary concept word, rare disease supplementary concept word, unique identifier, synonyms]
10. social perception*.mp. [mp=title, abstract, original title, name of substance word, subject heading word, keyword heading word, protocol supplementary concept word, rare disease supplementary concept word, unique identifier, synonyms]
11. exp Social Perception/
12. exp space perception/
13. physical space*.mp. [mp=title, abstract, original title, name of substance word, subject heading word, keyword heading word, protocol supplementary concept word, rare disease supplementary concept word, unique identifier, synonyms]
14. socio‐ecological.mp. [mp=title, abstract, original title, name of substance word, subject heading word, keyword heading word, protocol supplementary concept word, rare disease supplementary concept word, unique identifier, synonyms]
15. public place*.mp. [mp=title, abstract, original title, name of substance word, subject heading word, keyword heading word, protocol supplementary concept word, rare disease supplementary concept word, unique identifier, synonyms]
16. social context*.mp. [mp=title, abstract, original title, name of substance word, subject heading word, keyword heading word, protocol supplementary concept word, rare disease supplementary concept word, unique identifier, synonyms]
17. 5 or 6 or 7 or 8 or 9 or 10 or 11 or 12 or 13 or 14 or 15 or 16
18. 4 and 17
19. limit 18 to (humans and yr=“2007‐Current”)
